# Association Between the Genetic Risk for Attention-Deficit/Hyperactivity Disorder and Cognitive Function in Older Age: The MYHAT Population-Based Study

**DOI:** 10.1016/j.jagp.2025.10.005

**Published:** 2025-10-22

**Authors:** Douglas Teixeira Leffa, Yingjin Zhang, Yueting Wang, Esther G. Teverovsky, Erin Jacobsen, Bruna Bellaver, Pamela C.L. Ferreira, Kang-Hsien Fan, M. Ilyas Kamboh, Thomas K. Karikari, Chung-Chou H. Chang, Beth E. Snitz, Brooke S.G. Molina, Tharick A. Pascoal, Mary Ganguli

**Affiliations:** Department of Psychiatry, University of Pittsburgh School of Medicine, Pittsburgh, PA; Department of Biostatistics and Health Data Science, University of Pittsburgh School of Public Health, Pittsburgh, PA; Department of Biostatistics and Health Data Science, University of Pittsburgh School of Public Health, Pittsburgh, PA; Department of Psychiatry, University of Pittsburgh School of Medicine, Pittsburgh, PA; Department of Psychiatry, University of Pittsburgh School of Medicine, Pittsburgh, PA; Department of Psychiatry, University of Pittsburgh School of Medicine, Pittsburgh, PA; Department of Psychiatry, University of Pittsburgh School of Medicine, Pittsburgh, PA; Department of Human Genetics, University of Pittsburgh School of Public Health, Pittsburgh, PA; Department of Human Genetics, University of Pittsburgh School of Public Health, Pittsburgh, PA; Department of Psychiatry, University of Pittsburgh School of Medicine, Pittsburgh, PA; Department of Biostatistics and Health Data Science, University of Pittsburgh School of Public Health, Pittsburgh, PA; Department of Medicine, University of Pittsburgh School of Medicine, Pittsburgh, PA; Department of Neurology, University of Pittsburgh School of Medicine, Pittsburgh, PA; Departments of Psychiatry, Psychology, Pediatrics, Clinical and Translational Science, University of Pittsburgh, Pittsburgh, PA; Department of Psychiatry, University of Pittsburgh School of Medicine, Pittsburgh, PA; Department of Neurology, University of Pittsburgh School of Medicine, Pittsburgh, PA; Department of Neurology, University of Pittsburgh School of Medicine, Pittsburgh, PA; Department of Epidemiology, University of Pittsburgh School of Public Health, Pittsburgh, PA

**Keywords:** ADHD, cognitive function, older adults, polygenic risk scores, blood-based biomarkers, population-based cohort

## Abstract

**Objectives:**

Attention-deficit/hyperactivity disorder (ADHD) has been associated with increased risk for dementia, including Alzheimer’s disease (AD). Polygenic risk scores for ADHD (ADHD-PRS) reflect the genetic liability for the disorder and have been linked to cognitive decline in selected clinical samples. This study examined associations between ADHD-PRS and cognitive function in older adults from a population-based cohort and tested whether these associations were moderated by plasma biomarkers of AD and related disorders (ADRD).

**Methods:**

We analyzed data from 1,468 dementia-free participants from the Monongahela–Youghiogheny Healthy Aging Team (MYHAT) study, with cognitive assessments across five domains. ADHD-PRS was calculated, and a subset underwent blood draw for the analysis of ADRD biomarkers. We examined cross-sectional and longitudinal associations between ADHD-PRS and cognition using linear regressions and mixed-effects models adjusting for age, sex, and ancestry.

**Results:**

Higher ADHD-PRS was significantly associated with lower visuospatial function in cross-sectional analyses after correction for multiple comparisons. Associations with attention and language were moderated by education, and higher ADHD-PRS was associated with lower performance only among individuals with ≤ high school education. No associations were found between ADHD-PRS and plasma biomarkers, nor did biomarkers modify the relationship between ADHD-PRS and cognition. In longitudinal analyses, no association between ADHD-PRS and cognitive trajectories remained significant after correction for multiple comparisons.

**Conclusions:**

Overall, ADHD genetic liability appears relevant to cognitive performance in late life, though longitudinal trajectories remain unaffected. In older age, ADHD-PRS seems to negatively impact visuospatial tests, characterized by complex and integrative demands. Educational attainment moderated the associations with attention and language, suggesting a potential buffering effect.

## INTRODUCTION

Attention-deficit/hyperactivity disorder (ADHD) is a neurodevelopmental disorder characterized by impairing symptoms of inattention, hyperactivity-impulsivity, or both.^1^ It affects individuals throughout the lifespan, with a prevalence ranging from 5.3% in school-aged children^2^ to 1% in adults older than 60 years.^3^ ADHD is still under investigated in older age relative to younger populations.^4^ However, given the global demographic trend toward an aging population, the absolute number of older adults fulfilling the criteria for a diagnosis of ADHD is expected to increase.^5^ Therefore, elucidating the characteristics and impact of ADHD in older populations will be of growing importance.

Recent epidemiological studies relying primarily on electronic health records suggest that ADHD is associated with a higher risk for a diagnosis of mild cognitive impairment (MCI) and dementia of any cause.^6–9^ These associations persist despite adjustment for relevant confounders such as psychiatric comorbidities and metabolic disorders.^8,9^ A specific association between ADHD and Alzheimer’s Disease (AD) has been debated. On the one hand, AD is the most common cause of dementia,^10^ and roughly 60%–80% of individuals with a clinical diagnosis of dementia are found to have significant AD neuropathology at autopsy.^11–13^ Additionally, a large population-based study observed higher risk of AD among relatives of individuals with ADHD, indicating shared familial risk.^14^ On the other hand, prior studies reported no associations between ADHD and AD in analyses by dementia subtype after adjustment for confounders, suggesting a relationship that is not clearly specific to AD.^15–18^ An obstacle to studying age-related cognitive decline in ADHD is that the application of standard diagnostic criteria in older adults has been challenged.^4,19^ Additionally, many late-life studies lack data on the ADHD clinical phenotype. An alternative approach is to use a well-established genetic marker of ADHD, the ADHD polygenic risk score (ADHD-PRS), which represents the combined genetic liability for the disorder and is associated with ADHD diagnosis and related traits in independent samples.^20^

Recent research suggests that higher ADHD-PRS is associated with reduced resilience to age-related pathological abnormalities, including amyloid beta (A*β*) deposition.^21,22^ Additionally, in cognitively impaired individuals, higher ADHD-PRS was associated with impaired executive function, elevated tau pathology, as well as hypometabolism in frontal and parietal brain regions.^23^ Together, these findings suggest that ADHD-PRS may be a relevant factor influencing cognitive decline in older adults. However, previous studies investigating the association between ADHD-PRS and cognitive function have relied primarily on non-representative samples from participants enrolled in AD research at academic medical centers.^21,23^ Those individuals tend to come from higher socioeconomic backgrounds with substantial educational attainment, reflecting broader recruitment and retention biases common in AD research, particularly regarding genetic data provision.^24–26^ Overall, such biases raise concerns about the generalizability of findings to the broader population.

Therefore, the present study aims to investigate both cross-sectional and longitudinal associations between genetic risk for ADHD, measured with ADHD-PRS, and cognitive function within a population-based cohort. Furthermore, this study examined whether PRS-cognition associations vary according to baseline levels of plasma biomarkers previously linked to the pathology of AD and related disorders (ADRD) (A*β* [A*β*42], tau phosphorylated at threonine 217 [p-tau217], neurofilament light chain [NfL], and glial fibrillary acidic protein [GFAP]),^27^ testing the hypothesis that plasma biomarker will moderate the association between ADHD-PRS and cognition.

## METHODS

### Participants

We used data from the Monongahela–Youghiogheny Healthy Aging Team (MYHAT), a longitudinal, population based study aimed at identifying risk factors for MCI in a group of small town communities of relatively low socioeconomic status in southwestern Pennsylvania, USA. Participants were recruited using age stratified random sampling from publicly available voter registration lists for the 2004 and 2014 general elections. Recruitment took place in two waves using identical procedures: initially between 2006–2008 for participants aged 65 years and older (N = 2,036 screened), and subsequently between 2016–2019 for participants aged 65–74 years (N = 709 screened). Inclusion criteria for MYHAT at study entry were age 65 or older, living in one of the selected towns, not residing in long term care, sufficient hearing and vision to complete neuropsychological testing, and decisional capacity to provide informed consent. All screened individuals underwent the age- and education corrected Mini-Mental State Examination (MMSE), and those scoring <21 at study entry were classified as having substantial cognitive impairment and excluded from further assessment and follow-up. The remaining eligible participants underwent detailed baseline evaluations and were invited for annual follow-up assessments and optional blood and/or saliva collection at various timepoints (N = 2,685) ^28^. All recruitment and assessment procedures were approved by the University of Pittsburgh Institutional Review Board, and all participants provided written informed consent. For the current study, we included participants with baseline cognitive data and genome-wide genotyping (n = 1,468, Supplementary Fig. 1). For the current analyses, the baseline visit was defined as the visit closest to the date of plasma sample collection (for those with plasma data) or the visit with the most complete set of cognitive assessments (for those without plasma data). Based on the Clinical Dementia Rating (CDR),^29^ individuals with dementia at their baseline assessment (CDR ≥1) were excluded, leaving a cohort with CDR = 0 (normal cognition) or CDR = 0.5 (MCI). Participants with at least one annual follow-up visit were included in longitudinal analyses (N = 1,290). Depression symptoms were assessed using the modified Center for Epidemiological Studies Depression (mCES-D), and participants were categorized as mCES-D < 3 or mCES-D ≥3 (representing the 90^th^ percentile in the MYHAT cohort at study entry).^28^

### Polygenic Risk Scores

Genotyping was performed using two platforms: Platform Illumina HumanOmni1-Quad BeadChip (batch 1) and Platform Illumina Infinium Global Diversity Array-8 v1.0 (batch 2). Single nucleotide polymorphisms (SNPs) with a minor allele frequency <1%, locus missingness >5%, or Hardy-Weinberg equilibrium p < e-06 were excluded. Individuals with genotype missingness >5% or identity by descent $\hat{\pi }$ > 0.125 (closer than third-degree relatives) were also excluded. Genotypes were imputed using the Michigan Imputation Server, with batch 1 imputed using the 1000 Genomes Phase 3 panel and batch 2 using the HRC panel. SNPs with an imputation R^2^ < 0.3 were excluded. ADHD-PRS were computed using an additive model based on the most recent genome-wide association study (GWAS)^30^ and calculated with PRSice v2.2.^31^ Independent SNPs were identified using a 250-kb window and 0.1 r^2^ linkage disequilibrium criteria. After quality control, 269,728 variants remained. Eleven PRSs were computed using GWAS p-value thresholds of 1, 0.5, 0.4, 0.3, 0.2, 0.1, 0.05, 0.005, 0.0005, 5e-06, and 5e-08. Then, we performed principal-component analysis on these scores and used the first principal component as our aggregate ADHD-PRS. This approach has been empirically validated as effective and robust to quantify polygenic risk, reducing multiple testing and type I error.^23,32^ All PRSs were transformed into z-scores. To investigate populational structure, principal components analysis was conducted using PLINK 1.9,^33^ and the first ten principal components were used in all statistical models to control for ancestry.

### Cognitive Function

At baseline and at each subsequent annual assessment, participants underwent a comprehensive battery of neuropsychological tests designed to evaluate cognitive functioning across five domains: attention/psychomotor speed (Digit Span Forward, Trail Making Test A), executive function (Trail Making Test B, clock drawing, phonemic verbal fluency for letters P&S), memory (Wechsler Memory Scale-Revised Logical Memory [immediate and delayed recall], 12-item Face Name Associative Memory Exam, and Fuld Object Memory Evaluation), language (Boston Naming Test, semantic verbal fluency, and Indiana University Token Test), and visuospatial function (Wechsler Adult Intelligence Scale-III Block Design and Benton Visual Form Discrimination Test).^34^ To generate domain-specific composite scores, raw test scores were first converted into z-scores based on the baseline mean and standard deviation of the sample. Composite scores for each domain were then calculated by averaging the standardized scores of the available tests within that domain for individuals with at least one test score. Higher composite scores reflect better cognitive performance.

### Plasma Biomarkers

Blood specimens for plasma ADRD biomarker levels were drawn from participants starting in 2014, 8 years after initial cohort recruitment, by which time some attrition had already occurred. Biomarker levels were measured using single-molecule array (Simoa) technology on a Quanterix HD-X platform at the Department of Psychiatry, University of Pittsburgh School of Medicine. A*β*42, NfL and GFAP were quantified using the Neurology 4-Plex E assay while p-tau217 was analyzed using single-plex Simoa.^35^ All biomarker values were log-transformed to achieve approximate normality. In cross-sectional data, log A*β*42 was bimodal, so a two-component Gaussian mixture model was applied and yielded low (N = 83) and high (N = 536) clusters. Due to limited power, we restricted our modeling to data from the larger group. In longitudinal analyses, smaller samples produced a unimodal log A*β*42 distribution, and all log-transformed biomarkers were analyzed as continuous.

### Statistical Analysis

We used multiple linear regression models to examine the cross-sectional associations between ADHD-PRS and cognitive domain scores (attention, executive, language, memory, and visuospatial), adjusting for sex, age, and genetic ancestry (Model 1). To assess robustness and evaluate potential confounding or effect modification, we conducted a series of sensitivity analyses, detailed in the Supplementary Material. These included progressive adjustment for education (≤ versus > high school; Model 2), depression symptoms (Model 3), and vascular risk factors (VRFs) burden, measured using a composite score (Model 4, composite score specified in the Supplementary Material).^36,37^ We also tested education as a potential effect modifier by including an ADHD-PRS × education interaction term (Model 5). Associations between ADHD-PRS and plasma biomarkers were examined using linear regression models (Model 6), and biomarker effect modification was evaluated using ADHD-PRS × biomarker interaction terms (Model 7). For longitudinal analyses, we used linear mixed-effects models with random intercepts and slopes to test whether ADHD-PRS was associated with changes in cognitive domain scores over time. The primary longitudinal model included ADHD-PRS × time interactions and adjusted for age, sex, and ancestry (Model 8). As recommended for gene-environment interaction studies, we included interaction terms between ADHD-PRS and covariates when appropriate.^38^ To evaluate biomarker modification of longitudinal associations, we tested a three-way interaction term (ADHD-PRS × time × biomarker; Model 9). Full model specifications and VRF score methodology can be found in the Supplementary Material.

All statistical tests were two-sided with an alpha level of 0.05. The Benjamini–Hochberg procedure was used to control the false discovery rate (FDR) across cognitive domains for a total of five tests in each model and biomarker analyses for a total of four tests in each model. All analyses were conducted using R statistical software version 4.2.

## RESULTS

A total of 1,468 participants with both cognitive and genetic data at baseline and were included in the cross-sectional analyses. Among these, 678 had plasma ADRD biomarkers data collected within ±6 months of their annual cognitive assessment (Supplementary Table 1). The median age was 74 years (IQR = 69–82), 878 (59.8%) were women, 72 (4.9%) were non-White, and 758 (51.6%) had more than a high school education. Of the 1,468 participants, 1,290 had at least one follow-up assessment and were included in the longitudinal analyses, including 605 with available AD biomarker data. The mean follow-up duration was 6.37 years (SD = 4.42), with a median of 4.47 years (IQR = 3.42–8.98) and a maximum of 15 years. Descriptive statistics for baseline demographics, clinical characteristics, and cognitive measures are presented in Table 1. ADHD-PRS values were approximately normally distributed (Shapiro-Wilk normality test, W = 0.999, p = 0.478, Supplementary Fig. 2).

### Cross-Sectional Analyses

**FIGURE 1.**
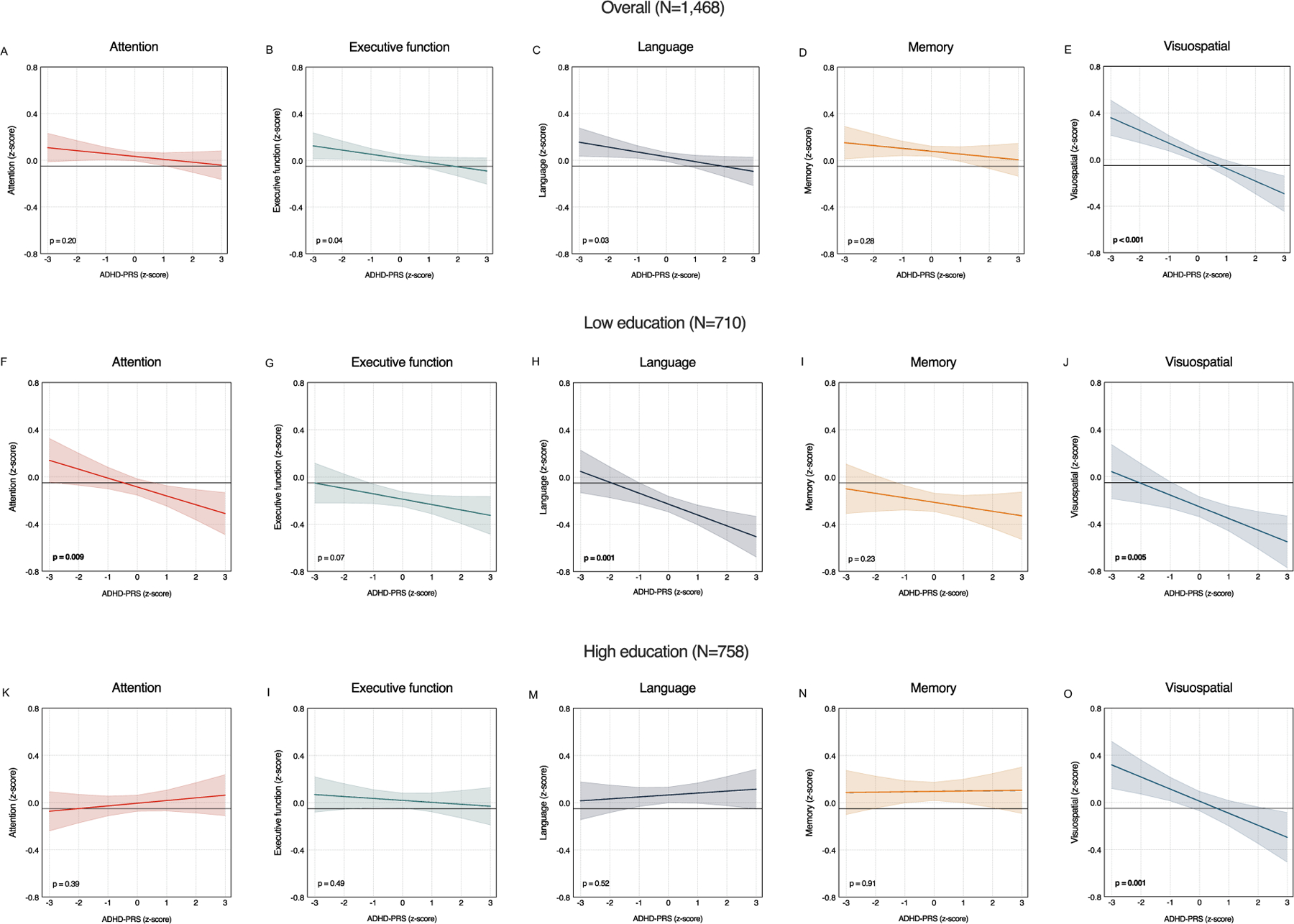
Cross-sectional associations between ADHD-PRS and cognitive domain scores. Associations between ADHD-PRS and cognitive domain scores were examined using multiple linear regression models. In the overall sample (Panels A–E), higher ADHD-PRS was significantly associated with lower visuospatial function (*t*[1335]=–4.461; *β* = −0.109, 95% CI = −0.157 to −0.061, p <0.001, FDR adjusted p <0.001; panel [E]), but not attention (*t*[1443] = −1.263; *β* = −0.025, 95% CI = −0.064 to 0.014, p = 0.207, FDR adjusted p = 0.276; panel [A]), executive function (*t*[1451] = −1.988; *β* = −0.036, 95% CI = −0.071 to −0.0004, p = 0.047, FDR adjusted p = 0.094; panel [B]), language (*t*[1443] = −2.128; *β* = −0.042, 95% CI = −0.080 to −0.003, p = 0.033, FDR adjusted p = 0.094; panel [C]), or memory (*t*[1438] = −1.077; *β* = −0.024, 95% CI = −0.069 to 0.020, p = 0.282, FDR adjusted p = 0.282; panel [D]). Panels [F–J] and [K–O] show associations within the lower (≤ high school) and higher (> high school) education groups, respectively. In the lower education group, higher ADHD–PRS was associated with lower attention (*t*[1440] = −2.632; *β* = −0.075, 95% CI = −0.131 to −0.019, p = 0.009, FDR adjusted p = 0.015; panel [F]), language (*t*[1440] = −3.337; *β* = −0.092, 95% CI = −0.146 to −0.038, p = 0.001, FDR adjusted p = 0.005; panel [H]), and visuospatial function (*t* [1332] = −2.811; *β* = −0.099, 95% CI = −0.168 to −0.030, p = 0.005, FDR adjusted p = 0.013; panel [J]), but not executive function (*t* [1448] = −1.765; *β* = −0.046, 95% CI = −0.096 to 0.005, p = 0.078, FDR adjusted p = 0.098; panel [G]), or memory (*t*[1435] = −1.182; *β* = −0.038, 95% CI = −0.101 to 0.025, p = 0.238, FDR adjusted p = 0.238; panel [I]). In the higher education group, higher ADHD-PRS was associated with lower visuospatial function (*t*[1332] = −3.203; *β* = −0.102, 95% CI = −0.165 to −0.040, p = 0.001, FDR adjusted p = 0.005; panel [O]), but not attention (*t*[1440] = 0.858; *β* = 0.023, 95% CI = −0.029 to 0.075, p = 0.391, FDR adjusted p = 0.650; panel [K]), executive function (*t*[1448] = −0.689; *β* = −0.016, 95% CI = −0.063 to 0.030, p = 0.491, FDR adjusted p = 0.650; panel [L]), language (*t*[1440] = 0.644; *β* = 0.016, 95% CI = −0.034 to 0.067, p = 0.520, FDR adjusted p = 0.650; panel [M]), or memory (*t*[1435] = 0.105; *β* = 0.003, 95% CI = −0.055 to 0.062, p = 0.916, FDR adjusted p = 0.916; panel [N]). Lines and shaded bands represent estimated marginal means and 95% confidence intervals (CIs) from linear regression models adjusted for covariates. P values in each panel are uncorrected and reflect the effect of ADHD-PRS on each cognitive domain; and values in bold indicate significance after controlling for multiple comparisons. The false discovery rate (FDR) was controlled using the Benjamini–Hochberg procedure for a total of five tests. Model specifications are provided in the Supplementary Material. ADHD-PRS = Attention-Deficit/Hyperactivity Disorder Polygenic Risk Score.

Higher ADHD-PRS was associated with lower executive function (Fig. 1B), language (Fig. 1C), and visuospatial function (Fig. 1E), but not attention (Fig. 1A) or memory (Fig. 1D). After controlling for multiple comparisons using the FDR, only the association with visuospatial function remained statistically significant (Fig. 1A–E and Supplementary Table 2). This association remained robust after additional adjustment for education, depressive symptoms, and VRFs (Supplementary Table 2).

Effect modification analyses showed significant ADHD-PRS × education interactions for attention and language (Supplementary Table 3), both of which remained significant after FDR correction. Stratified analyses showed that higher ADHD-PRS was associated with lower attention and lower language function among individuals with ≤ high school education, but not among those with > high school education (Fig. 1F–O and Supplementary Table 4). For visuospatial function, negative associations with ADHD-PRS were observed in both education strata (Fig. 1F–O and Supplementary Table 4).

ADHD-PRS was not significantly associated with plasma levels of A*β*42, p-tau217, NfL, or GFAP (Table 2). Additionally, no significant ADHD-PRS × biomarker interactions were found for any cognitive domain after FDR correction (Table 3).

### Longitudinal Analyses

**FIGURE 2.**
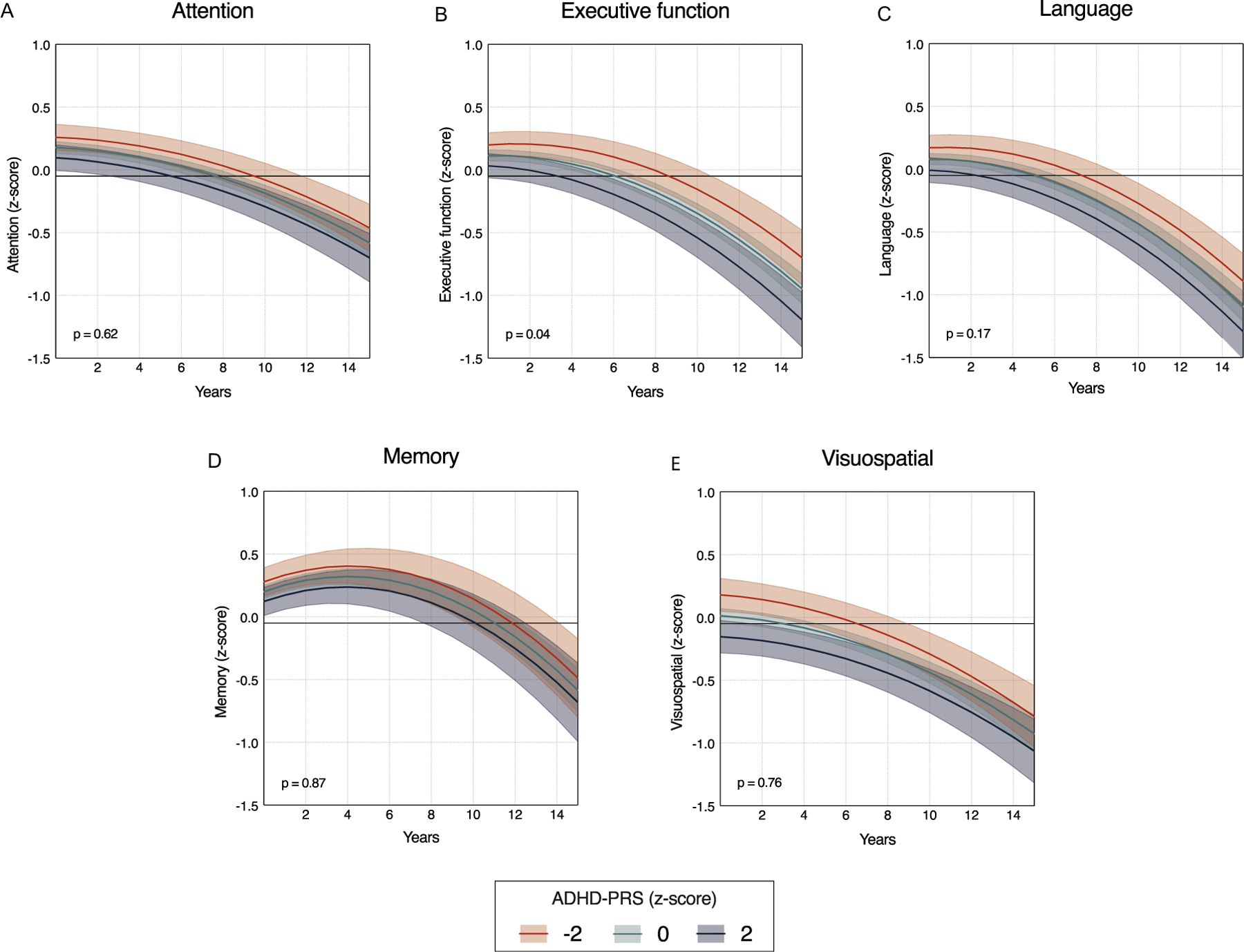
Longitudinal associations between ADHD-PRS and cognitive domain scores. Associations between ADHD-PRS and longitudinal changes in cognitive domain scores were examined using linear mixed-effects models with random intercepts and slopes. Higher ADHD-PRS was not significantly associated with changes over time in attention (ADHD-PRS × time; *t*[7463] = −0.488; *β* = −0.001, 95% CI = −0.006 to 0.003, p = 0.625, FDR adjusted p = 0.878; panel [A]), executive (*t*[7605] = −1.978; *β* = −0.005, 95% CI = −0.011 to −0.0001, p = 0.048, FDR adjusted p = 0.240; panel [B]), language (*t*[7482] = −1.343; *β* = −0.003, 95% CI = −0.009 to 0.001, p = 0.179, FDR adjusted p = 0.447; panel [C]), memory (*t*[7362] = −0.153; *β* = −0.0006, 95% CI = −0.008 to 0.007, p = 0.878, FDR adjusted p = 0.878; panel [D]), or visuospatial function (*t*[5286] = 0.298; *β* = 0.001, 95% CI = −0.005 to 0.007, p = 0.765, FDR adjusted p = 0.878; panel [E]), after correction for multiple comparisons. A nominal association with steeper decline in executive function was observed but did not remain significant after controlling for multiple comparisons using the Benjamini–Hochberg false discovery rate (FDR) procedure for a total of five tests. Lines and shaded bands represent estimated marginal means and 95% confidence intervals (CIs) from linear mixed-effects models with random intercepts and slopes, including ADHD-PRS × time interaction terms and adjustment for covariates. P values in each panel are uncorrected and indicate the significance of the ADHD-PRS × time interaction for each cognitive domain. Model specifications are detailed in the Supplementary Material. ADHD-PRS = Attention-Deficit/Hyperactivity Disorder Polygenic Risk Score.

In longitudinal analyses, we observed a significant ADHD-PRS × time interaction for executive function (Fig. 2B), suggesting that higher ADHD-PRS was associated with a greater rate of decline. However, this association did not remain significant after correction for multiple comparisons. No significant ADHD-PRS × time interactions were found for attention (Fig. 2A), language (Fig. 2C), memory (Fig. 2D), or visuospatial function (Fig. 2E). Additionally, no significant three-way interactions (ADHD-PRS × time × biomarker) were observed after FDR correction (Supplementary Table 6), indicating that baseline levels of plasma A*β*42, p-tau217, NfL, or GFAP levels did not significantly modify the longitudinal association between ADHD-PRS and cognitive trajectories.

## DISCUSSION

This study evaluated cross-sectional and longitudinal associations between ADHD-PRS and cognitive function in a population-based cohort of older adults. We additionally explored whether these associations were moderated by plasma biomarkers of AD pathology. Our primary finding was that higher ADHD-PRS was robustly associated with poorer visuospatial performance, a relationship that persisted after adjustment for education, depression symptoms, and vascular risk factors. Additionally, the associations between ADHD-PRS and attention and language were moderated by education, with higher ADHD-PRS associated with lower performance exclusively among participants with lower educational attainment (high school or less). We found no evidence that plasma AD biomarkers moderated the association between ADHD-PRS and cognitive performance or longitudinal cognitive trajectories.

In this representative sample of older adults, the most consistent finding was the association of ADHD-PRS with lower visuospatial function, measured by the Block Design and the Benton Visual Form Discrimination tests. These tasks involve comparison and manipulation of subtle shape and visual pattern differences, which may be particularly sensitive to disruptions in attention, planning, systematic scanning, and error checking. Indeed Kohs^39^ emphasized in the seminal introduction of the Block Design test that a successful performance demands focused attention, adaptation to novel situations, and critical evaluation of solutions. Previous research across the lifespan has consistently linked ADHD-PRS with cognitive deficits, particularly in attention and executive function,^20,30,40^ including among older adults.^21,41^ Our findings suggest that the complex and integrative demands of these visuospatial tests may specifically tap into cognitive processes disproportionately vulnerable to genetic ADHD risk in older age. The cross-sectional association between ADHD-PRS and visuospatial function was small in magnitude, yet in this cohort it exceeded the sizes of the corresponding associations for plasma A*β* and tau biomarkers,^35^ thus supporting its potential clinical relevance. To our knowledge, this study is the first to highlight this specific vulnerability in older adults.

The selective association with visuospatial performance is noteworthy because visuospatial deficits are among the earliest changes reported in Lewy body dementia and in Parkinson’s disease dementia.^42,43^ Prior studies have also linked ADHD to disorders within the Lewy body spectrum.^16,18,44^ Taken together, our results may indicate vulnerability of frontostriatal and posterior cortical networks that support visuospatial processing. We did not measure alpha synuclein or related markers and therefore cannot attribute this pattern to Lewy body pathology. However, this would be a noteworthy topic for future investigation.

Importantly, we observed a significant moderation effect by education, such that ADHD-PRS was associated with impaired attention and language performance only among individuals in the lower education attainment strata. This finding suggests that higher educational levels may protect against the adverse cognitive effects of genetic risk for ADHD in older populations. One plausible explanation involves cognitive reserve, as suggested by substantial evidence from AD research indicating that higher educational attainment is associated with resilience against cognitive decline.^45^ Given prior evidence of a genetic correlation between ADHD and lower educational attainment,^30,46^ individuals carrying higher ADHD genetic risk may be more likely to have lower education, placing them at compounded vulnerability. Thus, our findings illustrate not only the buffering role of cognitive reserve in mitigating genetically influenced cognitive deficits but also emphasize the complex interplay between genetic predisposition and environmental factors.

Although the cognitive profile of ADHD has been extensively studied in children and younger adults,^47^ research in older populations remains limited and presents mixed findings. For example, previous studies of older adults diagnosed with ADHD report deficits in attention, processing speed, episodic memory, working memory, and inhibition,^48,49^ while findings from population-based samples have been less consistent after controlling for depression symptoms.^50^ Our study contributes to this limited but growing body of research by identifying a specific link between ADHD genetic risk and visuospatial function in older adults, and it emphasizes a possible protective effect of educational attainment on attention and language. Developing standardized diagnostic criteria for ADHD in older adults, which remain absent, could facilitate improved recognition and more accurate characterization of cognitive profiles in aging ADHD populations.

We did not observe an association between ADHD-PRS and measurements of executive function or memory after adjusting for multiple comparisons. Similarly, we did not observe significant longitudinal associations between ADHD-PRS and cognitive decline over time. Although there was a nominal interaction with executive function, suggesting a decrease in executive function over time in those with higher ADHD-PRS, this did not meet the threshold for statistical significance after adjusting for multiple comparisons. These findings contrast with results from a study using data from the Alzheimer’s Disease Neuroimaging Initiative (ADNI), where higher ADHD-PRS was linked to persistent executive dysfunction among cognitively unimpaired individuals, and also with a decline in general cognition and memory over a six-year follow-up period.^21^ We emphasize that ADNI comprises a self-selected cohort of individuals motivated to enroll in dementia research. The discrepancy may therefore arise from differences in participant selection, as participants enrolled in AD research at academic medical centers, like those from ADNI, are typically from higher educational backgrounds and selected through stringent exclusion criteria, resulting in a relatively homogeneous sample with fewer comorbidities.^21,23–26^ In contrast, our sample was drawn from a community-based population. It might be speculated that, in more heterogeneous populations, the cognitive effects of ADHD-PRS are diluted by greater variability in baseline functioning and competing risk factors or comorbid conditions. Notably, the fact that we observed nominal associations with executive function and language before multiple-comparison adjustment supports the idea that the ADHD-PRS impact on lower cognitive performance may have been attenuated in this more heterogeneous sample.

Our results show that, in this representative sample of dementia-free individuals, ADHD-PRS was not significantly associated with plasma biomarkers previously linked to ADRD pathophysiology (A*β*42, p-tau217, NfL, and GFAP).^27^ We hypothesized that higher ADHD-PRS would be associated with higher plasma p-tau217, based on prior studies from our group,^21,23^ and that NfL and GFAP might also show associations given reported correlations among these biomarkers.^51^ Our finding supports the possibility that genetic risk for ADHD does not directly impact early stages of AD pathology when measured using plasma biomarkers.^15–17^ It is possible, however, that such associations might emerge at later disease stages or among individuals who have already developed clinical AD dementia, who typically exhibit higher levels of these biomarkers.^37^ In fact, prior studies showed higher levels of cerebrospinal fluid p-tau, but not A*β*, in dementia participants with higher ADHD-PRS.^23^ Additionally, we found no evidence that baseline plasma biomarker levels moderated the association between ADHD-PRS and cognitive performance in either cross-sectional or longitudinal analyses. These results contrast with earlier studies using positron emission tomography, which reported moderation effects of baseline A*β* burden on the relationship between ADHD-PRS and cognitive trajectories among cognitively unimpaired older adults.^21^ The observed differences could be explained by the use of plasma biomarkers in comparison to brain positron emission tomography,^52^ and also by the use of a population-based sample when compared to participants enrolled in AD research at academic medical centers.

Findings from this study should be interpreted with consideration of the following limitations. First, we used the ADHD-PRS as an indirect measure of genetic liability for the disorder, and ADHD symptomatology was not directly assessed. While ADHD-PRS is a validated and robust indicator of genetic risk, it may not fully capture the clinical phenotype of ADHD in older adults. Second, our neuropsychological battery included simple attention measures (Digit Span and Trail Making Test A) that capture psychomotor speed and simple working memory. These differ from more complex measures commonly used specifically for the assessment of ADHD, including the Continuous Performance Task,^53^ limiting the sensitivity to detect ADHD-related attentional deficits. Third, although the MYHAT is a population-based representative study, the cohort is predominantly White and drawn from a specific geographic region in southwestern Pennsylvania. Thus, generalizability to more ethnically or geographically heterogeneous populations may be limited. Fourth, although we adjusted for several vascular risk factors, we did not systematically adjust for all medical comorbidities that might influence cognitive outcomes, and residual confounding cannot be excluded. Fifth, plasma measures were available in a subsample that differed modestly from the rest of the cohort (younger, more women, higher education, lower MCI), which may reflect survival or participation bias. Sixth, neuropsychological tests are multidimensional, and any categorization of our tests into specific domains, although consistent with the literature, remains somewhat arbitrary. Finally, biomarker data were only measured one time. Therefore, we could not assess changes in biomarkers longitudinally or examine whether longitudinal changes in biomarkers might mediate or moderate changes in cognitive function related to ADHD-PRS.

To conclude, our findings highlight a specific association between the genetic liability for ADHD and impaired visuospatial function in older adults, independent of education, depression symptoms and VRFs. The moderating effect of education on attention and language performance underscores the potential protective role of cognitive reserve. Although we found no consistent evidence linking ADHD genetic risk to other cognitive domains or longitudinal cognitive decline, future studies should further investigate these relationships in diverse cohorts using multi-modal biomarkers and refined cognitive assessments to further clarify the impact of ADHD-PRS in cognitive function in older age.

## Supplementary Material

Supplemental file

Supplementary material associated with this article can be found in the online version at https://doi.org/10.1016/j.jagp.2025.10.005.

## Figures and Tables

**TABLE 1.** Baseline Demographics and Clinical Characteristics of the MYHAT Study Cohort

	Overall (N = 1,468)	With Longitudinal Data (N = 1,290)
Age, y, median (Q1, Q3)	74 (69, 82)	72 (68, 80)
Sex, No. (%)		
Women	878 (59.8%)	787 (61.0%)
Men	590 (40.2%)	503 (39.0%)
Race, No. (%)		
American Indian/Alaska Native	1 (0.1%)	1 (0.1%)
Asian	2 (0.1%)	2 (0.2%)
Black	65 (4.4%)	53 (4.1%)
More than one race	4 (0.3%)	2 (0.2%)
White	1,396 (95.1%)	1,232 (95.5%)
Education, No. (%)		
≤HS	710 (48.4%)	612 (47.4%)
>HS	758 (51.6%)	678 (52.6%)
Depressive symptoms		
mCES-D score, median (Q1, Q3)	0 (0, 1)	0 (0, 1)
<3 mCES-D score, No. (%)	1,267 (86.3%)	1,109 (86.1%)
≥3 mCES-D score, No. (%)	201 (13.7%)	179 (13.9%)
APOE ε4, No. carriers (%)	325 (22.2%)	284 (22.0%)
MMSE, median (Q1, Q3)	28 (26, 29)	28 (26, 29)
MCI, No. (%)	295 (20.1%)	237 (18.4%)
Attention, mean (SD)	0.04 (0.76)	0.10 (0.75)
Executive function, mean (SD)	0.02 (0.71)	0.07 (0.68)
Language, mean (SD)	0.03 (0.77)	0.08 (0.73)
Memory, mean (SD)	0.10 (0.86)	0.10 (0.76)
Visuospatial function, mean (SD)	0.03 (0.93)	0.10 (0.95)

	**Overall (N = 678)**	**With Longitudinal Data (N = 605)**

A*β*42, median (Q1, Q3)	6.78 (5.30, 8.13)	6.80 (5.24, 8.25)
p-tau217, pg/mL, median (Q1, Q3)	0.36 (0.25, 0.58)	0.36 (0.25, 0.59)
NfL, pg/mL, median (Q1, Q3)	21.60 (16.30, 33.12)	21.77 (16.27, 31.89)
GFAP, pg/mL, median (Q1, Q3)	125.45 (86.49, 186.91)	123.28 (86.26, 184.13)

*Notes:* Table 1 shows participant characteristics at baseline for the entire sample as well as the sample with longitudinal data. A total of 678 and 605 participants had plasma biomarker data for cross sectional and longitudinal analyses, respectively. Numbers of individuals with missing data for each variable are reported in the Supplementary Material. MCI was defined as CDR ≥0.5. Values are n (%) for categorical variables and mean (SD) or median (Q1, Q3) for continuous variables. Cognitive domain scores are z scores. MYHAT: Monongahela–Youghiogheny Healthy Aging Team; Q1, Q3: first and third quartiles; SD: standard deviation; HS: high school; mCES-D: modified Center for Epidemiological Studies Depression (range 0–20); APOE ε4: apolipoprotein E epsilon 4; MMSE: Mini-Mental State Examination; MCI: mild cognitive impairment; CDR: Clinical Dementia Rating; A*β*42: amyloid-*β* 42; p-tau217: phosphorylated tau 217; NfL: neurofilament light chain; GFAP: glial fibrillary acidic protein.

**TABLE 2.** Association Between ADHD-PRS and Plasma Biomarkers

Biomarker	B	SE	95% CI	t	N	DF	p value	FDR Adjusted p value
A*β*42	0.007	0.004	(−0.002, 0.015)	1.524	535	521	0.128	0.273
p-tau217	−0.013	0.009	(−0.032, 0.005)	−1.412	660	646	0.158	0.273
NfL	−0.004	0.007	(−0.018, 0.010)	−0.575	660	646	0.566	0.566
GFAP	−0.009	0.007	(−0.022, 0.005)	−1.270	660	646	0.205	0.273

*Notes:* Table 2 presents the associations between ADHD-PRS and plasma biomarkers. Analyses were performed using multiple linear regression models. Beta coefficients represent the effect of ADHD-PRS on each plasma biomarker level, adjusting for sex, age (years of age at baseline), and ancestry (first 10 principal components, Model 6, Supplementary Material). Positive beta values indicate a direct relationship where higher ADHD-PRS is associated with higher biomarker levels, while negative values indicate an inverse relationship. Statistical significance was determined at p < 0.05, with adjusted p-values controlling for FDR across all biomarkers for a total of 4 tests. ADHD-PRS: Attention-Deficit/Hyperactivity Disorder Polygenic Risk Score; *β*: beta coefficient; SE: standard error; CI: 95% confidence interval; t: t-statistic; DF: degrees of freedom; A*β*42: amyloid-beta 42; p-tau217: phosphorylated tau 217; NfL: neurofilament light chain; GFAP: glial fibrillary acidic protein; FDR: false discovery rate.

**TABLE 3.** Interaction Effects Between ADHD-PRS and Plasma Biomarkers on Domain-Specific Cognitive Composite Scores

Domain	β	SE	95% CI	t	N	DF	p value	FDR Adjusted p value
ADHD-PRS × A*β*42								
Attention	−0.083	0.045	(−0.173, 0.006)	−1.837	527	509	0.067	0.168
Executive	−0.047	0.040	(−0.126, 0.033)	−1.151	532	532	0.250	0.417
Language	−0.087	0.041	(−0.168, −0.005)	−2.090	528	510	0.037	0.168
Memory	−0.032	0.052	(−0.135, 0.072)	−0.601	524	506	0.548	0.685
Visuospatial	−0.011	0.053	(−0.116, 0.094)	−0.207	480	462	0.836	0.836
ADHD-PRS × p-tau217								
Attention	0.008	0.017	(−0.025, 0.042)	0.500	650	632	0.618	0.945
Executive	0.001	0.015	(−0.029, 0.031)	0.068	657	639	0.945	0.945
Language	0.009	0.015	(−0.021, 0.040)	0.600	650	632	0.549	0.945
Memory	0.045	0.020	(0.007, 0.083)	2.310	646	628	0.021	0.105
Visuospatial	0.003	0.021	(−0.039, 0.045)	0.138	569	551	0.890	0.945
ADHD-PRS × NfL								
Attention	−0.005	0.018	(−0.040, 0.030)	−0.276	650	632	0.783	0.783
Executive	−0.008	0.016	(−0.039, 0.023)	−0.499	657	639	0.618	0.783
Language	−0.010	0.016	(−0.042, 0.022)	−0.624	650	632	0.533	0.783
Memory	−0.007	0.021	(−0.048, 0.034)	−0.325	646	628	0.746	0.783
Visuospatial	0.016	0.024	(−0.031, 0.064)	0.674	566	548	0.501	0.783
ADHD-PRS × GFAP								
Attention	0.023	0.020	(−0.016, 0.061)	1.151	650	632	0.250	0.597
Executive	0.009	0.018	(−0.026, 0.045)	0.529	657	639	0.597	0.597
Language	0.023	0.018	(−0.013, 0.058)	1.260	650	632	0.208	0.597
Memory	−0.017	0.023	(−0.062, 0.028)	−0.727	646	628	0.468	0.597
Visuospatial	0.016	0.025	(−0.033, 0.066)	0.644	566	548	0.520	0.597

*Notes:* Table 3 presents the interaction effects between ADHD-PRS and plasma biomarkers on cognitive performance across five domains. Analyses were performed using multiple linear regression models. Beta coefficients represent the effect of the interaction term (ADHD-PRS × plasma biomarker) on each cognitive domain score, controlling for sex, age (years of age at baseline), ancestry (first 10 principal components), education, and depressive symptoms (Model 7, Supplementary Material). Positive beta values indicate that the association between ADHD-PRS and cognitive performance becomes more positive (or less negative) as biomarker levels increase. Statistical significance was determined at p < 0.05, with adjusted p-values controlling for FDR across all cognitive domains for a total of 5 tests. Sample sizes vary across analyses depending on available data for each biomarker and cognitive domain. ADHD: Attention-Deficit/Hyperactivity Disorder; PRS: Polygenic Risk Score; *β*: beta coefficient; SE: standard error; CI: 95% confidence interval; t: t-statistic; DF: degrees of freedom; A*β*42: amyloid-beta 42; p-tau217: phosphorylated tau 217; NfL: neurofilament light chain; GFAP: glial fibrillary acidic protein; FDR: false discovery rate.

## Data Availability

De-identified participant data are available upon reasonable request to qualified investigators with an approved IRB protocol and a signed Data Use Agreement using the Contact Us option at www.dementia-epidemiology.pitt.edu.

## Highlights

- What is the primary question addressed by this study?

Does the genetic risk for ADHD, measured with polygenic risk scores (ADHD-PRS), impact cognitive function in older adults from a population-based cohort, and is this effect influenced by Alzheimer’s disease biomarkers?

- What is the main finding of this study?

Higher ADHD-PRS was significantly associated with poorer visuospatial function in older adults, independently of education, depression, and vascular risk factors. Educational attainment moderated the association between ADHD-PRS and attention and language functions, with negative associations observed in individuals with lower education. Results were not modified by Alzheimer’s disease biomarkers.

- What is the meaning of the finding?

ADHD-PRS seems to negatively impact visuospatial tests in older age, characterized by complex and integrative demands. Results support education as a potential protective factor against the impact of ADHD-PRS in attention and language functions in older age.
