# Supplementary material for: Association Between the Genetic Risk for Attention-Deficit/Hyperactivity Disorder and Cognitive Function in Older Age: The MYHAT Population-Based Study
Source: Am J Geriatr Psychiatry. Author manuscript; Available in PMC 2026 Jan 21. (PMC12817166; doi:10.1016/j.jagp.2025.10.005)
Supplement: Supplemental file [file NIHMS2123997-supplement-Supplemental_file.docx]

**SUPPLEMENTARY MATERIAL: TABLE OF CONTENTS**

[Statistical Models and Notation 3](#_Toc208384952)

[Study Sampling 5](#_Toc208384953)

[Missing data 7](#_Toc208384954)

[Supplementary Table 1. Baseline characteristics by availability of plasma biomarker data. 8](#_Toc208384955)

[Supplementary Table 2. Association between ADHD-PRS and domain-specific cognitive composite scores 11](#_Toc208384956)

[Supplementary Table 3. Interaction effects between ADHD-PRS and education on domain-specific cognitive composite scores 13](#_Toc208384957)

[Supplementary Table 4. ADHD-PRS effects on domain-specific cognitive composite scores by education level 14](#_Toc208384958)

[Supplementary Table 5. Interaction effects between ADHD-PRS and time on domain-specific cognitive composite scores 15](#_Toc208384959)

[Supplementary Table 6. Interaction effects between ADHD-PRS, time and plasma biomarkers on domain-specific cognitive composite scores 16](#_Toc208384960)

[Supplementary Table 7. Baseline medication use and nicotine exposure. 18](#_Toc208384961)

[Supplementary Figure 1. Flow-chart 19](#_Toc208384962)

[Supplementary Figure 2. ADHD-PRS has a normal distribution in the studied population 20](#_Toc208384963)

[Construction of the Vascular Risk Factor Score 21](#_Toc208384964)

[Joint Modeling Framework 21](#_Toc208384965)

[Variable Definitions and Inclusion Criteria 21](#_Toc208384966)

[Model Fitting and Variable Selection 22](#_Toc208384967)

[Composite Score Construction 22](#_Toc208384968)

[Model Validation Using Composite Scores 22](#_Toc208384969)

[Distribution and Interpretation of Composite Scores 23](#_Toc208384970)

[Supplementary Table 8. Definition and categorization of variables considered for model selection including baseline and time-varying variables considered for inclusion in the joint model 24](#_Toc208384971)

[Supplementary Table 9. Baseline characteristics of study participants 25](#_Toc208384972)

[Supplementary Table 10. Parameter estimates from initial joint model of MCI risk includes all available vascular/metabolic predictors and APOE4 status 27](#_Toc208384973)

[Supplementary Table 11. Parameter estimates from final joint model of MCI risk includes selected vascular/metabolic predictors and APOE4 status after backward stepwise selection 28](#_Toc208384974)

[Supplementary Table 12. Association between vascular risk composite score and MCI risk from joint model 29](#_Toc208384975)

[Supplementary Table 13. Distribution of vascular composite scores by MCI status 30](#_Toc208384976)

[Supplementary Figure 3. Density plot of vascular composite score between MCI and non-MCI groups 31](#_Toc208384977)

[Supplementary Figure 4. Vascular risk factor composite score contribution 32](#_Toc208384978)

# Statistical Models and Notation

i. $\beta_{0}$ denotes the fixed intercept;

ii. ${ADHD\_PRS}_{i}$ represents the ADHD-PRS for subject $i$;

iii. ${Sex}_{i}$ is a binary indicator for sex (1 = female, 0 = male) for subject $i$;

iv. ${Age}_{i}$is the baseline age (in years) for subject $i$;

v. ${Ancestry}_{ki}$ refers to the $k$th ancestry principal component ($k=1, \ldots, 10$, indicating the first 10 principal components) for subject $i$, included to account for any ancestry differences in genetic structure that could bias the results;

vi. ${Education}_{i}$ is a binary indicator for educational attainment (1 = more than high school, 0 = high school or less) for subject $i$;

vii. ${Depressive symptoms}_{i}$ is a binary indicator of baseline depressive symptom burden (1 = mCES-D ≥ 3, 0 = mCES-D < 3) for subject $i$;

viii. ${Biomarker}_{i}$ denotes the baseline plasma biomarker level for subject $i$;

ix. ${VRFs}_{i}$ denotes vascular risk factors burden for subject $i$;

x. ${Time}_{ij}$ represents the time in years (continuous variable) for subject $i$ at study time point $j$;

xi. $\varepsilon_{i}$ is the residual error term in linear regression models for subject $i$;

xii. $\upsilon_{0i}$ represents the subject-specific random intercept in linear mixed-effects models;

xiii. $\varepsilon_{ij}$ is the residual error terms for subject $i$ at time point $j$*.*

Model 1:

$${Cognitive function}_{i}=\beta_{0}+\beta_{1}{ADHD\_PRS}_{i}+\beta_{2}{Sex}_{i}+\beta_{3}{Age}_{i}+\sum_{k=1}^{10} \beta_{4k}{Ancestry}_{ki}+\varepsilon_{i}$$

Model 2:

$${Cognitive function}_{i}=\beta_{0}+\beta_{1}{ADHD\_PRS}_{i}+\beta_{2}{Sex}_{i}+\beta_{3}{Age}_{i}+\sum_{k=1}^{10} \beta_{4k}{Ancestry}_{ki}+ \beta_{5}{Education}_{i}+ \varepsilon_{i}$$

Model 3:

$${Cognitive function}_{i}=\beta_{0}+\beta_{1}{ADHD\_PRS}_{i}+\beta_{2}{Sex}_{i}+\beta_{3}{Age}_{i}+\sum_{k=1}^{10} \beta_{4k}{Ancestry}_{ki}+ \beta_{5}{Education}_{i}+ \beta_{6}{Depressive symptoms}_{i}+ \varepsilon_{i}$$

Model 4:

${Cognitive function}_{i}=$ $\beta_{0}+\beta_{1}{ADHD\_PRS}_{i}+\beta_{2}{Sex}_{i}+\beta_{3}{Age}_{i}+\sum_{k=1}^{10} \beta_{4k}{Ancestry}_{ki}+ \beta_{5}{Education}_{i}+ \beta_{6}{Depressive symptoms}_{i}+ \beta_{7}{VRFs}_{i}+ \varepsilon_{i}$

Model 5:

$${Cognitive function}_{i}=\beta_{0}+{\beta_{1}{ADHD\_PRS}_{i} X {Education}_{i}+ \beta}_{2}{ADHD\_PRS}_{i}+\beta_{3}{Sex}_{i}+\beta_{4}{Age}_{i}+\sum_{k=1}^{10} \beta_{5k}{Ancestry}_{ki}+ \beta_{6}{Education}_{i}+ \beta_{7}{Depressive symptoms}_{i}+ \varepsilon_{i}$$

Model 6:

$${Plasma biomarker}_{i}=\beta_{0}+\beta_{1}{ADHD\_PRS}_{i}+\beta_{2}{Sex}_{i}+\beta_{3}{Age}_{i}+\sum_{k=1}^{10} \beta_{4k}{Ancestry}_{ki}+\varepsilon_{i}$$

Model 7:

$${Cognitive function}_{i}=\beta_{0}+{\beta_{1}{ADHD\_PRS}_{i}\times{\beta_{2}Biomarker}_{i}+\beta_{2}{Biomarker}_{i}+ \beta}_{3}{ADHD\_PRS}_{i}+\beta_{4}{Sex}_{i}+\beta_{5}{Age}_{i}+\sum_{k=1}^{10} \beta_{6k}{Ancestry}_{ki}+ \beta_{7}{Education}_{i}+ \beta_{8}{Depressive symptoms}_{i}+ \varepsilon_{i}$$

Model 8:

$${Cognitive function}_{i}=\beta_{0}+\beta_{1}{ADHD\_PRS}_{i}\times{Time}_{ij}+\beta_{2}{ADHD\_PRS}_{i}+\beta_{3}{Time}_{ij}+\beta_{4}{ADHD\_PRS}_{i}\times{Sex}_{i}+\beta_{5}{Time}_{ij}\times{Sex}_{i}+\beta_{6}{Sex}_{i}+\beta_{7}{ADHD\_PRS}_{i}\times{Age}_{i}+\beta_{8}{Time}_{ij}\times{Age}_{i}+\beta_{9}{Age}_{i}+\sum_{k=1}^{10} \beta_{10k}{Ancestry}_{ki}+\upsilon_{0i}+\varepsilon_{ij}$$

Model 9:

$${Cognitive function}_{i}=\beta_{0}+\beta_{1}{ADHD\_PRS}_{i} \times{Time}_{ij}\times{Biomarker}_{i}+\beta_{2}{ADHD\_PRS}_{i}+\beta_{3}{Time}_{ij}+{\beta_{4}Biomarker}_{i}+\beta_{5}{ADHD\_PRS}_{i}\times{Sex}_{i}+\beta_{6}{Time}_{ij}\times{Sex}_{i}+\beta_{7}{Biomarker}_{i}\times{Sex}_{i}+\beta_{8}{Sex}_{i}+\beta_{9}{ADHD\_PRS}_{i}\times{Age}_{i}+\beta_{10}{Time}_{ij}\times{Age}_{i}+{\beta_{11}{Biomarker}_{i}\times{Age}_{i}+\beta}_{12}{Age}_{i}+\sum_{k=1}^{10} \beta_{13k}{Ancestry}_{ki}+\upsilon_{0i}+\varepsilon_{ij}$$

# Study Sampling

Sampling for the various analyses reported here was as follows. The total sample recruited by random sampling from the voter registration list was 2,745 (2,036 in 2006-2008, and 709 in 2016-2019). After excluding participants with an age- and education-corrected MMSE score < 21, the fully assessed participants numbered 1,982 in the original cohort and 703 in the replenishment cohort, for a total of 2,685.

Assessment at baseline and at annual follow-up visits included neuropsychological (cognitive) testing and Clinical Dementia Rating (CDR), among other items. All participants were requested to provide a blood sample by venipuncture or finger-prick (first recruitment cohort only) or a saliva sample (second recruitment cohort only) for DNA extraction and APOE genotyping. In the first cohort, 995 participants refused or were unable to provide blood samples or opted to provide finger-prick sample only. Those who provided finger-prick samples only did not have enough blood for GWAS. In the second recruitment cohort, 164 participants refused, or specimen-collection attempts were unsuccessful. A total of 1,482 participants had GWAS available at the time of this analysis.

Over the course of follow-up, some participants died, dropped out because of illness, relocated from the study area, or withdrew voluntarily. Typically, the older and less healthy individuals were most likely to be lost to follow-up. Starting in 2014, participants in the first recruitment cohort were requested to provide additional venipuncture samples, which were banked and later used for assays of plasma ADRD biomarkers. By this time, 1,118 participants had already left the study, leaving 864 active; 388 of these refused or were unable to provide blood specimens. Beginning in 2019, these participants were asked for another plasma sample. At that time, only 224 of the original cohort remained in the study, and 123 refused or could not provide a sample. Across both time frames, a total of 480 participants provided at least one plasma sample.

Participants in the second recruitment cohort were asked to provide blood samples for ADRD biomarkers at study entry and again at a follow-up visit beginning in 2019. Over that time frame, 269 participants refused or were unable to provide a sample, leaving a total of 440 participants with at least one blood sample for ADRD biomarkers. In total, 920 specimens were identified for ADRD biomarker assays; however, some samples had insufficient volume, were lost in storage, or had levels below assay detection limits. A total of 901 participants had complete data on all biomarkers reported here.

A total of 702 participants had plasma biomarker data and GWAS data. For plasma biomarker analyses, 683 participants had plasma assessments with collection dates occurring within six months of a study visit and were deemed eligible for inclusion. Participants were subsequently excluded for missing race information (n=1), missing modified Center for Epidemiologic Studies Depression Scale (mCES-D) scores (n=1), or CDR ≥ 1 (dementia) (n=3), yielding an analytic sample of 678 participants for plasma biomarker analyses.

Cross-sectional cognitive analyses employed different visit selection criteria based on plasma biomarker availability. For participants with eligible plasma biomarker data (n=683), we used the same analytic sample as the plasma analyses (n=678). For participants without plasma biomarker data or with ineligible data (n=799), cognitive scores were obtained from the earliest available visit with the most complete domain-specific cognitive composite score data. This approach was used because ADHD-PRS represents a time-invariant genetic measure, permitting cross-sectional analysis at any time point while maximizing statistical power through optimal data completeness. Among participants without plasma data, additional exclusions included those with dementia at the selected visit (n=8) and those missing all cognitive assessments (n=1), resulting in an analytic sample of 790 participants. Combined with the 678 participants from the plasma biomarker group, the final analytic sample for cross-sectional cognitive analyses comprised 1,468 participants.

For the longitudinal analyses, 1,290 participants had one or more years of follow-up since study entry and had PRS and repeated cognitive data; 605 of them had plasma biomarker data.

# Missing data

For the cross-sectional analyses (N=1,468), missing data were as follows: 3 individuals for APOE ε4 status, 3 for MMSE score, 11 for attention, 3 for executive function, 11 for language, 16 for memory, and 119 for visuospatial function. Among the 678 participants with biomarker data, the number of individuals missing each specific biomarker was: 60 for Aβ42, 18 for p-tau217, NfL, and GFAP.

For the longitudinal analyses using wave 1 as baseline (N=1,290), missing data were as follows: 3 participants for APOE ε4 status, 2 for attention, 1 for executive function, 1 for language, 2 for memory, 39 for visuospatial function, and 2 for mCES-D scores. For the longitudinal analyses using biomarker data wave as baseline (N=605), at biomarker data wave, missing data were as follows: 1 participant for APOE ε4 status, 9 for attention, 3 for executive function, 10 for language, 13 for memory, 84 for visuospatial function, and 2 for mCES-D scores. Among the 605 participants with biomarker data, the number of individuals missing each specific biomarker was: 63 for the Aβ42, 18 for p-tau217, and 21 for both NfL and GFAP.

# Supplementary Table 1. Baseline characteristics by availability of plasma biomarker data.

|  | Overall (N=1,468) | No plasma (N=790) | With plasma (N=678) | Test statistic | *P value* |
| --- | --- | --- | --- | --- | --- |
| Age, y, median (Q1, Q3) | 74 (69, 82) | 76 (70, 83) | 73 (69, 80) | W=224336 | < 0.001 |
| Sex, No. (%) |  |  |  | χ^2^(1)=8.618 | 0.003 |
| Women | 878 (59.8%) | 445 (56.3%) | 433 (63.9%) |  |  |
| Men | 590 (40.2%) | 345 (43.7%) | 245 (36.1%) |  |  |
| Race, No. (%) |  |  |  | - | 0.503 |
| American Indian/Alaska Native | 1 (0.1%) | 1 (0.1%) | 0 (0.0%) |  |  |
| Asian | 2 (0.1%) | 2 (0.3%) | 0 (0.0%) |  |  |
| Black | 65 (4.4%) | 38 (4.8%) | 27 (4.0%) |  |  |
| More than one race | 4 (0.3%) | 3 (0.4%) | 1 (0.1%) |  |  |
| White | 1,396 (95.1%) | 746 (94.4%) | 650 (95.9%) |  |  |
| Education, No. (%) |  |  |  | χ^2^(1)=8.663 | < 0.001 |
| ≤HS | 710 (48.4%) | 437 (55.4%) | 273 (40.3%) |  |  |
| >HS | 758 (51.6%) | 353 (44.7%) | 405 (59.7%) |  |  |
| Depressive symptoms |  |  |  | χ^2^(1)= 0.032 | 0.859 |
| <3 mCES-D score, No. (%) | 1,267 (86.3%) | 683 (86.5%) | 584 (86.1%) |  |  |
| ≥3 mCES-D score, No. (%) | 201 (13.7%) | 107 (13.5%) | 94 (13.9%) |  |  |
| APOE ε4, No. carriers (%) | 325 (22.2%) | 179 (22.7%) | 146 (21.6%) | χ^2^(1)=0.27897 | 0.597 |
| MMSE, median (Q1, Q3) | 28 (26, 29) | 27 (26, 29) | 28 (27, 29) | W=312176 | <0.001 |
| MCI, No. (%) | 295 (20.1%) | 205 (25.9%) | 90 (13.3%) | χ^2^(1)=36.506 | < 0.001 |
| Attention, mean (SD) | 0.04 (0.76) | 0.01 (0.75) | 0.08 (0.76) | F(1,1455)=2.960 | 0.086 |
| Executive function, mean (SD) | 0.02 (0.71) | -0.06 (0.71) | 0.13 (0.69) | F(1,1463)=25.90 | < 0.001 |
| Language, mean (SD) | 0.03 (0.77) | -0.05 (0.81) | 0.12 (0.72) | F(1,1455)=17.545 | < 0.001 |
| Memory, mean (SD) | 0.10 (0.86) | -0.05 (0.80) | 0.27 (0.90) | F(1,1450)=53.527 | < 0.001 |
| Visuospatial function, mean (SD) | 0.03 (0.93) | -0.05 (0.98) | 0.13 (0.86) | F(1,1347)=12.046 | < 0.001 |

Supplementary Table 1 shows participant characteristics at baseline for the overall sample and for subgroups without and with plasma biomarker data. Of the 1,468 participants overall, 678 provided plasma for cross-sectional analyses, and 605 of these had longitudinal cognitive follow-up. Values are n (%) for categorical variables and mean (SD) or median (Q1, Q3) for continuous variables. Cognitive domain scores are z scores. MCI was defined as CDR ≥ 0.5.

Abbreviations: MYHAT = Monongahela–Youghiogheny Healthy Aging Team; Q1, Q3 = first and third quartiles; SD = standard deviation; HS = high school; mCES-D = modified Center for Epidemiological Studies Depression (range 0–20); APOE ε4 = apolipoprotein E epsilon 4; MMSE = Mini-Mental State Examination; MCI = mild cognitive impairment; CDR = Clinical Dementia Rating.

# Supplementary Table 2. Association between ADHD-PRS and domain-specific cognitive composite scores

| **Domain** | **β** | **SE** | **95% CI** | **t** | **N** | **DF** | **P-value** | **FDR adjusted p-value** |
| --- | --- | --- | --- | --- | --- | --- | --- | --- |
| **Model 1** | | | | | | | | |
| **Attention** | -0.025 | 0.020 | (-0.064, 0.014) | -1.263 | 1457 | 1443 | 0.207 | 0.276 |
| **Executive** | -0.036 | 0.018 | (-0.071, -0.0004) | -1.988 | 1465 | 1451 | 0.047 | 0.094 |
| **Language** | -0.042 | 0.020 | (-0.080, -0.003) | -2.128 | 1457 | 1443 | 0.033 | 0.094 |
| **Memory** | -0.024 | 0.023 | (-0.069, 0.020) | -1.077 | 1452 | 1438 | 0.282 | 0.282 |
| **Visuospatial** | -0.109 | 0.024 | (-0.157, -0.061) | -4.461 | 1349 | 1335 | **<0.001** | **<0.001** |
| **Model 2** | | | | | | | | |
| **Attention** | -0.023 | 0.020 | (-0.061, 0.016) | -1.158 | 1457 | 1442 | 0.247 | 0.329 |
| **Executive** | -0.030 | 0.018 | (-0.065, 0.005) | -1.689 | 1465 | 1450 | 0.091 | 0.182 |
| **Language** | -0.034 | 0.019 | (-0.072, 0.003) | -1.787 | 1457 | 1442 | 0.074 | 0.182 |
| **Memory** | -0.017 | 0.022 | (-0.060, 0.027) | -0.742 | 1452 | 1437 | 0.458 | 0.458 |
| **Visuospatial** | -0.102 | 0.024 | (-0.149, -0.054) | -4.215 | 1349 | 1334 | **<0.001** | **<0.001** |
| **Model 3** | | | | | | | | |
| **Attention** | -0.022 | 0.020 | (-0.061, 0.016) | -1.141 | 1457 | 1441 | 0.254 | 0.339 |
| **Executive** | -0.030 | 0.018 | (-0.065, 0.005) | -1.689 | 1465 | 1449 | 0.091 | 0.182 |
| **Language** | -0.034 | 0.019 | (-0.071, 0.004) | -1.770 | 1457 | 1441 | 0.077 | 0.182 |
| **Memory** | -0.016 | 0.022 | (-0.059, 0.027) | -0.718 | 1452 | 1436 | 0.473 | 0.473 |
| **Visuospatial** | -0.101 | 0.024 | (-0.148, -0.054) | -4.226 | 1349 | 1333 | **<0.001** | **<0.001** |
| **Model 4** | | | | | | | | |
| **Attention** | -0.014 | 0.02 | (-0.053, 0.024) | -0.719 | 1457 | 1440 | 0.473 | 0.631 |
| **Executive** | -0.017 | 0.018 | (-0.052, 0.018) | -0.942 | 1465 | 1448 | 0.346 | 0.631 |
| **Language** | -0.019 | 0.019 | (-0.056, 0.018) | -0.99 | 1457 | 1440 | 0.323 | 0.631 |
| **Memory** | -0.010 | 0.022 | (-0.053, 0.034) | -0.432 | 1452 | 1435 | 0.666 | 0.666 |
| **Visuospatial** | -0.090 | 0.024 | (-0.137, -0.043) | -3.744 | 1349 | 1332 | **<0.001** | **<0.001** |

Supplementary Table 2 presents the associations between standardized ADHD-PRS z-score and cognitive performance across five domains. Analyses were performed using multiple linear regression models. Beta coefficients represent the effect of ADHD-PRS on each cognitive domain score, controlling for sex, age (years of age at baseline), and ancestry (first 10 principal components, Model 1, Supplementary Material). We also progressively adjusted for education (Model 2, Supplementary Material). depressive symptoms (Model 3, Supplementary Material), and VRFs (Model 4, Supplementary Material). Negative beta values indicate an inverse relationship, where higher ADHD-PRS is associated with lower cognitive scores. Statistical significance was determined at p < 0.05, with adjusted p-values controlling for FDR across all cognitive domains for a total of 5 tests within each model. P-values in bold indicate those that remained significant after controlling for multiple comparisons.

Abbreviations: ADHD = attention-deficit/hyperactivity disorder; PRS = polygenic risk score; β = beta coefficient; SE = standard error; CI = confidence interval; t = statistic for t test; DF = degrees of freedom; VRFs = vascular risk factors; FDR = false discovery rate.

# Supplementary Table 3. Interaction effects between ADHD-PRS and education on domain-specific cognitive composite scores

| **Domain** | **β** | **SE** | **95% CI** | **t** | **N** | **DF** | **P-value** | **FDR adjusted p-value** |
| --- | --- | --- | --- | --- | --- | --- | --- | --- |
| **ADHD-PRS × Education** | | | | | | | | |
| **Attention** | 0.098 | 0.039 | (0.022, 0.174) | 2.538 | 1457 | 1440 | **0.011** | **0.027** |
| **Executive** | 0.029 | 0.035 | (-0.039, 0.098) | 0.835 | 1465 | 1448 | 0.404 | 0.505 |
| **Language** | 0.109 | 0.037 | (0.035, 0.182) | 2.912 | 1457 | 1440 | **0.004** | **0.020** |
| **Memory** | 0.041 | 0.043 | (-0.044, 0.127) | 0.947 | 1452 | 1435 | 0.344 | 0.505 |
| **Visuospatial** | -0.003 | 0.047 | (-0.096, 0.089) | -0.068 | 1349 | 1332 | 0.946 | 0.946 |

Supplementary Table 3 presents the effect modifications by education level on the associations between standardized ADHD-PRS z-score and domain-specific cognitive composite scores. Analyses were performed using multiple linear regression models. Beta coefficients represent the effect of the interaction term (ADHD-PRS x education) on each domain, controlling for sex, age (years of age at baseline), ancestry (first 10 principal components), education, and depressive symptoms (Model 5, Supplementary Material). Positive beta values indicate that the association between ADHD PRS and cognitive performance differs by education level, with a stronger positive association (or weaker negative association) among those with higher education (>HS) compared to those with lower education (≤HS). Statistical significance was determined at p < 0.05, with adjusted p-values controlling for FDR across all cognitive domains for a total of 5 tests. P-values in bold indicate those that remained significant after controlling for multiple comparisons.

Abbreviations: ADHD = attention-deficit/hyperactivity disorder; PRS = polygenic risk score; β = beta coefficient; SE = standard error; CI = confidence interval; t = statistic for t test; DF = degrees of freedom; HS = high school; FDR = false discovery rate.

# Supplementary Table 4. ADHD-PRS effects on domain-specific cognitive composite scores by education level

| **Domain** | **Stratum** | **β** | **SE** | **95% CI** | **t** | **DF** | **P-value** | **FDR adjusted p-value** |
| --- | --- | --- | --- | --- | --- | --- | --- | --- |
| **Attention** | <= HS | -0.075 | 0.029 | (-0.131, -0.019) | -2.632 | 1440 | **0.009** | **0.015** |
| **Attention** | > HS | 0.023 | 0.026 | (-0.029, 0.075) | 0.858 | 1440 | 0.391 | 0.650 |
| **Executive** | <= HS | -0.046 | 0.026 | (-0.096, 0.005) | -1.765 | 1448 | 0.078 | 0.098 |
| **Executive** | > HS | -0.016 | 0.024 | (-0.063, 0.030) | -0.689 | 1448 | 0.491 | 0.650 |
| **Language** | <= HS | -0.092 | 0.028 | (-0.146, -0.038) | -3.337 | 1440 | **0.001** | **0.005** |
| **Language** | > HS | 0.016 | 0.026 | (-0.034, 0.067) | 0.644 | 1440 | 0.520 | 0.650 |
| **Memory** | <= HS | -0.038 | 0.032 | (-0.101, 0.025) | -1.182 | 1435 | 0.238 | 0.238 |
| **Memory** | > HS | 0.003 | 0.030 | (-0.055, 0.062) | 0.105 | 1435 | 0.916 | 0.916 |
| **Visuospatial** | <= HS | -0.099 | 0.035 | (-0.168, -0.030) | -2.811 | 1332 | **0.005** | **0.013** |
| **Visuospatial** | > HS | -0.102 | 0.032 | (-0.165, -0.040) | -3.203 | 1332 | **0.001** | **0.005** |

Supplementary Table 4 presents the associations between standardized ADHD-PRS z-score and cognitive performance by education level across five cognitive domains. Analyses were performed using multiple linear regression models. Beta coefficients represent the effect of ADHD PRS on each cognitive domain score within each education stratum (≤HS and >HS), controlling for ancestry, age, sex, and depressive symptoms. Negative beta values indicate an inverse relationship, where higher ADHD PRS is associated with lower cognitive scores. Statistical significance was determined at p < 0.05, with adjusted p-values controlling for FDR across all cognitive domains and strata, for a total of 5 tests within each education strata. P-values in bold indicate those that remained significant after controlling for multiple comparisons.

Abbreviations: ADHD = attention-deficit/hyperactivity disorder; PRS = polygenic risk score; β = beta coefficient; SE = standard error; CI = confidence interval; t = statistic for t test; DF = degrees of freedom; HS = high school education; FDR = false discovery rate.

# Supplementary Table 5. Interaction effects between ADHD-PRS and time on domain-specific cognitive composite scores

| **Domain** | **β** | **SE** | **95% CI** | **t** | **N** | **DF** | **P-value** | **FDR adjusted p-value** |
| --- | --- | --- | --- | --- | --- | --- | --- | --- |
| **ADHD-PRS × time** | | | | | | | | |
| **Attention** | -0.001 | 0.002 | (-0.006, 0.003) | -0.488 | 8756 | 7463 | 0.625 | 0.878 |
| **Executive** | -0.005 | 0.002 | (-0.011, -0.0001) | -1.978 | 8899 | 7605 | 0.048 | 0.240 |
| **Language** | -0.003 | 0.002 | (-0.009, 0.001) | -1.343 | 8776 | 7482 | 0.179 | 0.447 |
| **Memory** | -0.0006 | 0.004 | (-0.008, 0.007) | -0.153 | 8655 | 7362 | 0.878 | 0.878 |
| **Visuospatial** | 0.001 | 0.003 | (-0.005, 0.007) | 0.298 | 6542 | 5286 | 0.765 | 0.878 |

Supplementary Table 5 presents the interaction between ADHD-PRS and time on domain-specific cognitive scores, adjusting for sex, age (years of age at baseline), ancestry (first 10 principal components), and a time-squared term. Analyses were performed using linear mixed-effects models with random intercepts and slopes. Each model also adjusted to the interaction term between each adjusted covariate with time, and that with ADHD-PRS (Model 8, Supplementary Material). The beta coefficient represents the estimate of the effect of the interaction between standardized ADHD-PRS z-score and time. It is presented as the expected increase in yearly change in domain-specific cognitive score due to per 1-unit increase in ADHD-PRS z-score. Statistical significance was determined at p < 0.05, with adjusted p-values controlling for FDR across all cognitive domains, for a total of 5 tests.

Abbreviations: ADHD = attention-deficit/hyperactivity disorder; PRS = polygenic risk score; β = beta coefficient; SE = standard error; CI = confidence interval; t = statistic for t test; N = number of observations; DF = degrees of freedom.

# Supplementary Table 6. Interaction effects between ADHD-PRS, time and plasma biomarkers on domain-specific cognitive composite scores

| **Biomarker covariate** | **Cognitive Domain** | **β** | **SE** | **95% CI** | **t** | **N** | **DF** | **P-value** | **FDR adjusted p-value** |
| --- | --- | --- | --- | --- | --- | --- | --- | --- | --- |
| **ADHD-PRS × time × Biomarker** | | | | | | | | | |
| **Aβ42** | Attention | –0.009 | 0.022 | (–0.054, 0.035) | –0.397 | 2314 | 1773 | 0.690 | 0.863 |
|  | Executive | 0.025 | 0.019 | (–0.013, 0.063) | 1.278 | 2356 | 1810 | 0.201 | 0.335 |
|  | Language | 0.027 | 0.018 | (–0.007, 0.063) | 1.547 | 2316 | 1776 | 0.122 | 0.305 |
|  | Memory | 0.040 | 0.024 | (–0.008, 0.088) | 1.617 | 2272 | 1735 | 0.105 | 0.305 |
|  | Visuospatial | 0.004 | 0.030 | (–0.055, 0.063) | 0.131 | 1451 | 974 | 0.895 | 0.895 |
| **p-tau217** | Attention | -0.012 | 0.013 | (-0.038, 0.014) | -0.898 | 2569 | 1983 | 0.368 | 0.750 |
|  | Executive | -0.013 | 0.011 | (-0.036, 0.008) | -1.205 | 2617 | 2026 | 0.228 | 0.750 |
|  | Language | 0.001 | 0.010 | (-0.019, 0.022) | 0.130 | 2573 | 1988 | 0.896 | 0.905 |
|  | Memory | 0.009 | 0.013 | (-0.015, 0.035) | 0.754 | 2517 | 1935 | 0.450 | 0.750 |
|  | Visuospatial | 0.001 | 0.014 | (-0.025, 0.029) | 0.119 | 1598 | 1084 | 0.905 | 0.905 |
| **NfL** | Attention | -0.013 | 0.015 | (-0.044, 0.018) | -0.816 | 2573 | 1990 | 0.414 | 0.653 |
|  | Executive | 0.021 | 0.013 | (-0.004, 0.047) | 1.645 | 2619 | 2031 | 0.100 | 0.500 |
|  | Language | -0.003 | 0.012 | (-0.028, 0.022) | -0.231 | 2575 | 1993 | 0.817 | 0.817 |
|  | Memory | 0.020 | 0.015 | (-0.011, 0.051) | 1.263 | 2520 | 1941 | 0.206 | 0.516 |
|  | Visuospatial | 0.011 | 0.018 | (-0.024, 0.047) | 0.639 | 1592 | 1082 | 0.522 | 0.653 |
| **GFAP** | Attention | -0.010 | 0.015 | (-0.041, 0.020) | -0.668 | 2573 | 1990 | 0.504 | 0.630 |
|  | Executive | 0.023 | 0.013 | (-0.003, 0.049) | 1.716 | 2619 | 2031 | 0.086 | 0.215 |
|  | Language | 0.002 | 0.012 | (-0.022, 0.026) | 0.173 | 2575 | 1993 | 0.862 | 0.862 |
|  | Memory | 0.031 | 0.015 | (0.001, 0.062) | 2.040 | 2520 | 1941 | **0.041** | 0.207 |
|  | Visuospatial | 0.013 | 0.018 | (-0.022, 0.048) | 0.730 | 1592 | 1082 | 0.465 | 0.630 |

Supplementary Table 6 presents the interaction between ADHD-PRS, biomarker and time on domain-specific cognitive scores, adjusting for sex, age (years of age at baseline), ancestry (first 10 principal components), and a time-squared term. Analyses were performed using linear mixed-effects models with random intercepts and slopes. Each model includes also adjusted to the interaction term between each adjusted covariate with time, and that with ADHD-PRS (Model 9, Supplementary Material). The beta coefficient represents the estimate of the effect of the 3-way interaction of standardized ADHD PRS z-score, the included biomarker covariate, and time. Statistical significance was determined at p < 0.05, with adjusted p-values controlling for FDR across all cognitive domains, for a total of 5 tests within each biomarker group.

Abbreviations: ADHD = attention-deficit/hyperactivity disorder; PRS = polygenic risk score; β = beta coefficient; SE = standard error; CI = confidence interval; t = statistic for t test; N = number of observations; DF = degrees of freedom, Aβ42 = amyloid-beta 42; p-tau217 = phosphorylated tau 217; NfL = neurofilament light chain; GFAP = glial fibrillary acidic protein; FDR = false discovery rate.

# Supplementary Table 7. Baseline medication use and nicotine exposure.

| **Variable** | **Yes, n (%)** | **No, n (%)** | **Missing, n** |
| --- | --- | --- | --- |
| Antidepressant | 252 (17.2) | 1,214 (82.8) | 2 |
| Stimulant | 6 (0.4) | 1,460 (99.6) | 2 |
| Anti-anxiety medication | 71 (4.8) | 1,395 (95.2) | 2 |
| Antipsychotic | 16 (1.1) | 1,450 (98.9) | 2 |
| Mood stabilizer | 10 (0.7) | 1,456 (99.3) | 2 |
| Dementia/cognitive medication | 25 (1.7) | 1,441 (98.3) | 2 |
| Ever smoked (lifetime) | 792 (54.0) | 676 (46.0) | 0 |
| Smoked in the past year | 126 (8.6) | 1342 (91.4) | 0 |
| Currently smoking | 115 (7.8) | 1353 (92.2) | 0 |
| Cigars or pipe in past year | 27 (1.8) | 1,435 (98.2) | 6 |
| Chewing tobacco in past year | 21 (1.4) | 1,439 (98.6) | 8 |
| E-cigarette in past year | 9 (1.5) | 574 (98.5) | 885 |
| Nicotine patch or gum | 14 (2.4) | 569 (97.6) | 885 |

Supplementary Table 7 presents baseline medication use and nicotine exposure. Counts are shown for the full cohort at the defined baseline. Values are n (%) for Yes and No, with percentages calculated using the non-missing denominator for each variable. Medication use and nicotine exposure were self-reported at the study visit. Questions on e-cigarette use and nicotine patch or gum were added in 2016 and later discontinued, which accounts for the larger amount of missing data for those items.

# Supplementary Figure 1. Flow-chart


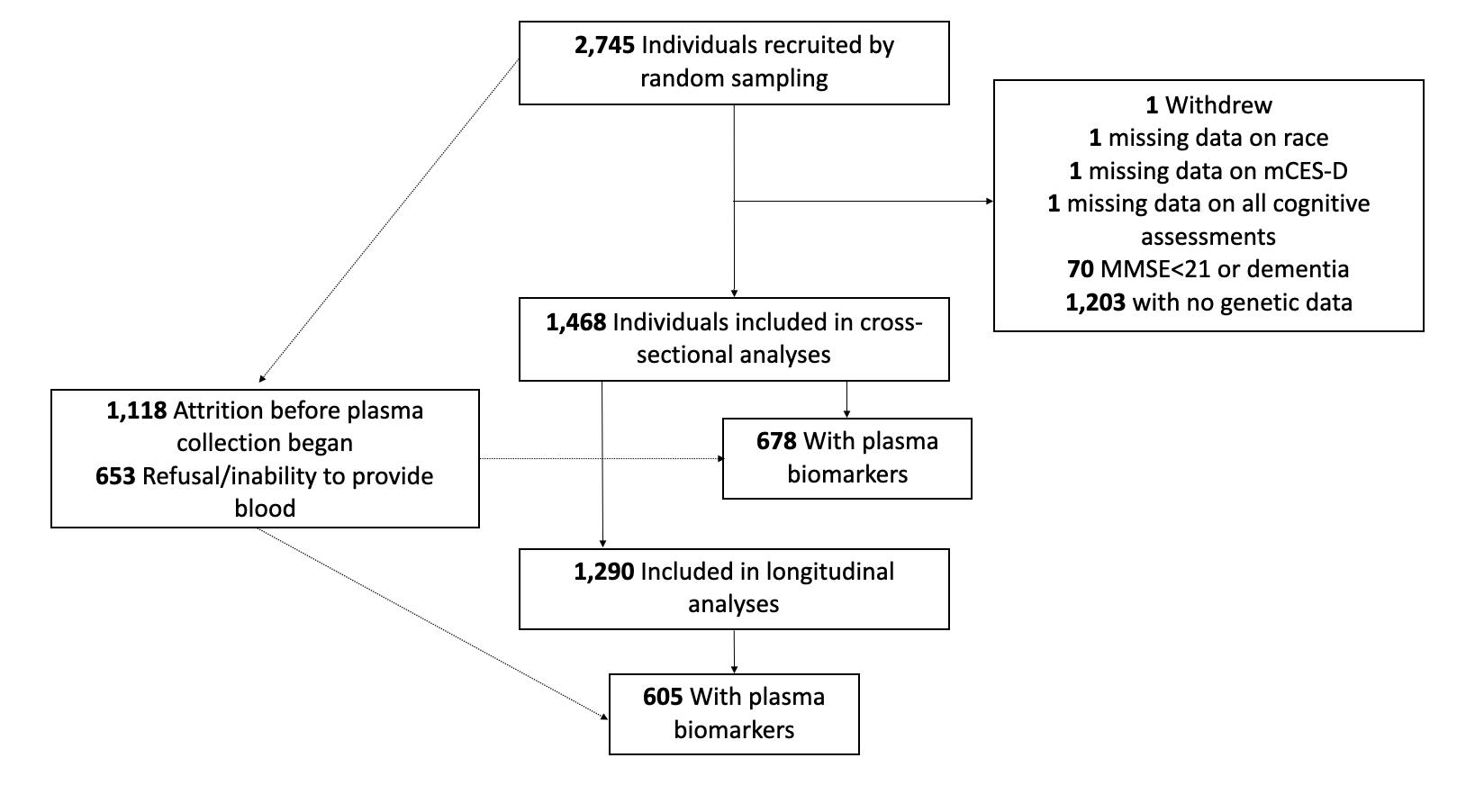


# Supplementary Figure 2. ADHD-PRS has a normal distribution in the studied population


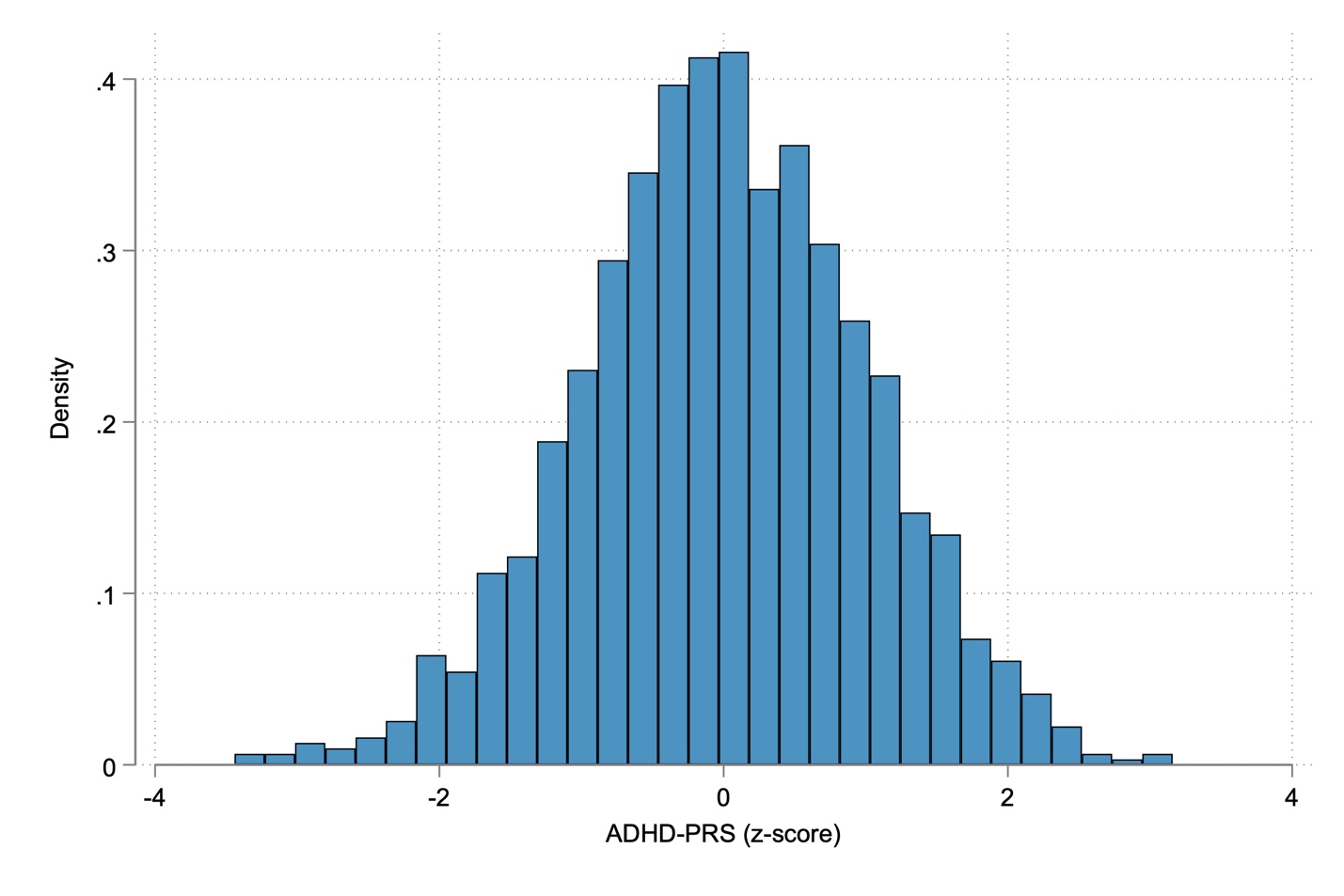


Abbreviations: ADHD = attention-deficit/hyperactivity disorder; PRS = polygenic risk score.

# Construction of the Vascular Risk Factor Score

This section describes the construction of the vascular risk factor (VRF) score. It was initially designed to predict the risk of mild cognitive impairment (MCI), using data from the MYHAT longitudinal cohort study. The goal was to derive a clinically interpretable score that summarizes contributions from vascular and metabolic risk factors.

## Joint Modeling Framework

We used a joint modeling approach consisting of two submodels:

1. Longitudinal submodel: A generalized linear mixed model (GLMM) with a binomial distribution and logit link function to model binary cognitive status (Clinical Dementia Rating [CDR] ≥ 0.5 for MCI vs. CDR = 0 for cognitive normal) across 10 study waves.
2. Dropout submodel: A Weibull proportional hazards model to account for informative dropout due to death or illness-related attrition.

These two submodels were linked through shared random effects. Both submodels were adjusted for age, sex, education, and APOE4 carrier status.

## Variable Definitions and Inclusion Criteria

Baseline definitions and distributions of all candidate predictors are summarized in Supplementary Table 8 and Supplementary Table 9, respectively.

- Outcome: MCI (CDR ≥ 0.5) vs. cognitively normal (CDR = 0).
- Covariates: Age (65-74, 75-84, ≥85 years), sex (male/female), education (<, =, or > high school), and APOE4 carrier status (yes/no).
- Time-varying variables:
  - Systolic blood pressure (SBP ≥120 vs. <120 mmHg, measured at the study visit with standard procedures)
  - Diastolic blood pressure (DBP ≥70 vs. <70 mmHg, measured at the study visit with standard procedures)
  - History of stroke or TIA, diabetes, heart failure, hypertension, coronary heart disease (CHD), cardiac arrhythmia, myocardial infarction, high cholesterol (obtained by self-report)
  - Smoking status (never vs. previous or current smoker)
  - Drinking status (never vs. past or current drinker).
- Baseline only
  - BMI (<25, 25-29, ≥30, derived from measured height and weight when available and from self-reported values otherwise).

## Model Fitting and Variable Selection

In order to construct composite scores, we followed these steps:

1. All predictors, including demographic covariates, were categorized using clinically meaningful thresholds. Reference levels were coded as 0.
2. We fit an initial joint model including all vascular/metabolic predictors and APOE4 status.
3. Reference categories were redefined so that all non-reference levels reflected increased MCI risk.

Results from the initial joint model described in Step 2 above are presented in Supplementary Table 10. Significant risk factors included the history of diabetes, hypertension, cardiac arrhythmia, and low DBP (<70 mmHg). Protective factors included overweight/obese BMI (≥25) and alcohol consumption (past or current). We then applied backward stepwise selection to remove predictors with weak associations (p > 0.05), continue until the largest p-value among vascular/metabolic variables was no greater than that of the demographic covariates. The final joint model (Supplementary Table 11) retained the following predictors: history of diabetes, hypertension, and cardiac arrhythmia, DBP (<70 or ≥70 mmHg), BMI (<25, 25-29, or ≥30), and APOE4 carrier status.

## Composite Score Construction

We derived a vascular composite score by translating estimated coefficients from the final model into integer-based weights. The smallest non-zero coefficient was scaled to 1, and all others were scaled proportionally and rounded to the nearest integer. The final score was calculated as a sum of weighted, dummy-coded predictor indicators. The composite score was defined as:

**Composite Score** = **2**×{age 75-84} + **10**×{age ≥85} + **1**×{male} + **2**×{HS education} + **9**×{<HS education} + **3**×{APOE4 carrier} + **1**×{previous/current smoker} + **2**×{never drinker} + **2**×{BMI 25-29} + **5**×{BMI <25} + **2**×{stroke/ TIA} + **3**×{diabetes} + **1**×{hypertension} + **1**×{cardiac arrhythmia} + **1**×{DBP <70}

Each term indicates the prevalence (1) or absence (0) of the respective risk factor. The total score reflects the cumulative burden of vascular and metabolic risk factors.

## Model Validation Using Composite Scores

We validated the composite score by fitting a model that replaced individual vascular predictors with the variable of composite scores while adjusting for demographics. Results (Supplementary Table 12) confirmed that higher composite scores were significantly associated with increased MCI risk.

## Distribution and Interpretation of Composite Scores

Each participant’s record was classified as MCI or non-MCI based on visit-specific status; a participant could contribute to both groups across visits. Supplementary Table 13 summarizes the distribution of composite scores (mean, median, range, quartiles) by MCI status. Supplementary Figure 3 presents kernel density plots, showing that MCI observations tend to have higher composite scores than non-MCI observations.

To explore the composition of total scores, we grouped records into three score categories: 0-9, 10-19, and ≥20. Supplementary Figure 4 shows radar charts depicting the relative contribution of each risk factor to the average composite score within each group. Greater variation in contributions from diabetes, hypertension, low DBP, and never drinking was observed across score groups, particularly among individuals with MCI.

## Supplementary Table 8. Definition and categorization of variables considered for model selection including baseline and time-varying variables considered for inclusion in the joint model

| **Category** | **Variables** |
| --- | --- |
| **Outcome (binary for GLMM)** | MCI (CDR ≥0.5 vs. 0) |
| **Adjusted covariate** | Demographics: Age (65-74, 75-84, ≥85), Sex, Education (<high school, =high school, >high school).  Biomarker: APOE4 Carrier |
| **Vascular /metabolic variables** | History of stroke or TIA, diabetes, congestive heart failure (CHF), hypertension, coronary heart disease (CHD), cardiac arrhythmia, myocardial infarction, high cholesterol,  systolic blood pressure (SBP ≥120 vs. <120 mmHg), diastolic blood pressure (DBP <70 vs. ≥70 mmHg),  Smoking status (never vs. previous or current smoker),  Drinking status (never vs. past or current drinker)  Baseline body mass index (BMI <25, 25-29, ≥30) |

## Supplementary Table 9. Baseline characteristics of study participants

| **Variable** | **Descriptive statistics**  **(N=1,716)** | **Notation** |
| --- | --- | --- |
| **Outcome** |  |  |
| **MCI (CDR ≥0.5), n (%)** | 457 (26.63) |  |
| **Adjusting covariates** |  |  |
| **Age (in years), mean (SD)** | 77.45 (7.36) |  |
| **Age (in years), median (Q1, Q3)** | 78 (71, 83) |  |
| **Age group** |  |  |
| **65-74, n (%)** | 605 (35.26) |  |
| **75-84, n (%)** | 794 (46.27) |  |
| **≥85, n (%)** | 317 (18.47) |  |
| **Sex (female), n (%)** | 1065 (62.06) |  |
| **Education group** |  |  |
| **<High School, n (%)** | 228 (13.29) |  |
| **=High School, n (%)** | 775 (45.16) |  |
| **>High School, n (%)** | 713 (41.55) |  |
| **APOE4 (carrier), n (%)** | 332 (19.35) |  |
| **Vascular /metabolic variables** |  |  |
| **Stroke and/or TIA, n (%)** | 155 (9.05) |  |
| **Missing, n** | 3 |  |
| **Diabetes, n (%)** | 337 (21.98) |  |
| **Missing, n** | 1 |  |
| **Congestive heart failure (CHF), n (%)** | 335 (19.53) | Myocardial infarction and/or congestive heart failure. |
| **Missing, n** | 1 |  |
| **Hypertension, n (%)** | 1117 (65.21) |  |
| **Missing, n** | 3 |  |
| **Coronary heart disease (CHD), n (%)** | 449 (26.21) | Cardiac catheterization and/or coronary bypass surgery. |
| **Missing, n** | 3 |  |
| **Cardiac arrhythmia, n (%)** | 507 (29.56) | Irregular heartbeat and/or pacemaker. |
| **Missing, n** | 1 |  |
| **Myocardial infarction, n (%)** |  |  |
| **Missing, n** |  |  |
| **High cholesterol, n (%)** | 1048 (61.32) |  |
| **Missing, n** | 7 |  |
| **SBP, mean (SD)** | 133.29 (15.07) |  |
| **SBP ≥120 mmHg, n (%)** | 1445 (84.70) |  |
| **Missing, n** | 10 |  |
| **DBP, mean (SD)** | 74.29 (9.37) |  |
| **DBP ≥70 mmHg, n (%)** | 1278 (74.96) |  |
| **Missing, n** | 10 |  |
| **Smoking (Previous or current), n (%)** | 898 (52.48) |  |
| **Missing, n** | 5 |  |
| **Drinking (Previous or current), n (%)** | 1471 (85.77) |  |
| **Missing, n** | 1 |  |
| **BMI, mean (SD)** | 28.43 (5.60) |  |
| **Underweight or normal (BMI <25), n (%)** | 551 (32.11) |  |
| **Overweight (25≤ BMI <30), n (%)** | 654 (38.11) |  |
| **Obesity (BMI ≥30), n (%)** | 511 (29.78) |  |

Abbreviations: SD, standard deviation; MCI, mild cognitive impairment; APOE4, Apolipoprotein E-ε4; SBP, systolic blood pressure; DBP, diastolic blood pressure; TIA, transient ischemic attack; BMI, body mass index.

Note: Mean and standard deviation values were reported for continuous variables as mean (SD) alone with median (Q1, Q3) if given skewed distribution, while frequency and relative frequency (%) out of non-missing observations were reported for categorical variables as n (%). Number of missing observations were reported as “N Missing”.

## Supplementary Table 10. Parameter estimates from initial joint model of MCI risk includes all available vascular/metabolic predictors and APOE4 status

| **Category** | **Variable** | **Estimate** | **SE** | **P-value*** | **Odds ratio (OR)** |
| --- | --- | --- | --- | --- | --- |
| **Intercept** | Intercept | -1.8806 | 0.4163 | <0.0001 | 0.152 |
| **APOE4** | Carrier | 0.4826 | 0.3198 | 0.1315 | 1.620 |
| **Vascular/metabolic**  **variables** | Stroke and/or TIA | 0.2842 | 0.2141 | 0.1846 | 1.329 |
|  | Diabetes | 0.7722 | 0.2107 | 0.0003 | 2.165 |
|  | CHF | 0.0729 | 0.2392 | 0.7604 | 1.076 |
|  | Hypertension | -0.2675 | 0.0988 | 0.0069 | 0.765 |
|  | CHD (cardiac catheterization and/or coronary bypass surgery) | -0.2155 | 0.1840 | 0.2418 | 0.806 |
|  | Hypertension (by history) | 0.5522 | 0.1439 | 0.0001 | 1.737 |
|  | Myocardial infarction | 0.0327 | 0.2409 | 0.8922 | 0.806 |
|  | Cardiac arrhythmia (irregular heartbeat and/or wear pacemaker) | 0.3188 | 0.1396 | 0.0226 | 1.375 |
|  | High cholesterol | -0.0376 | 0.1366 | 0.7833 | 0.963 |
|  | Low SBP (<120 mmHg) | -0.1832 | 0.1174 | 0.1188 | 0.833 |
|  | Low DBP (<70 mmHg) | -0.2675 | 0.0988 | 0.0069 | 0.765 |
|  | Previous or current smoking | 0.1080 | 0.2585 | 0.6762 | 1.114 |
|  | Previous or current drinking | -0.7105 | 0.3577 | 0.0471 | 0.491 |
|  | Underweight or normal BMI (<25) |  |  |  |  |
|  | Overweight (BMI 25-20) | -0.9480 | 0.3058 | 0.0020 | 0.388 |
|  | Obesity (BMI ≥30) | -1.8180 | 0.3292 | <0.0001 | 0.162 |

*A significance level of 0.05 was used; and statistically significant *p* values are shown in bold.

Abbreviations: CHD, coronary heart disease; CHF, congestive heart failure; DBP, diastolic blood pressure; SBP, systolic blood pressure.

## Supplementary Table 11. Parameter estimates from final joint model of MCI risk includes selected vascular/metabolic predictors and APOE4 status after backward stepwise selection

| **Category** | **Variable** | **Score** | **Estimate** | **SE** | **P-value** | **Odds ratio (OR)** |
| --- | --- | --- | --- | --- | --- | --- |
| **Intercept** | Intercept | / | -5.5969 | 0.3743 | <0.0001 | 0.004 |
| **Age** | Age 65-74 | 0 |  |  |  |  |
|  | Age 75-84 | 2 | 0.5373 | 0.1625 | 0.0010 | 1.711 |
|  | Age ≥85 | 10 | 2.2496 | 0.2025 | <0.0001 | 9.484 |
| **Sex** | Female | 0 |  |  |  |  |
|  | Male | 1 | 0.3159 | 0.2541 | 0.2139 | 1.371 |
| **Education** | higher than HS education | 0 |  |  |  |  |
|  | HS education | 2 | 0.5342 | 0.2559 | 0.0370 | 1.706 |
|  | lower than HS education | 9 | 2.0761 | 0.3692 | <0.0001 | 7.973 |
| **APOE4** | Carrier | 3 | 0.5982 | 0.2938 | 0.0419 | 1.819 |
| **Vascular**  **variable** | Stroke and/or TIA | 2 | 0.4143 | 0.2132 | 0.0521 | 1.513 |
|  | Diabetes | 3 | 0.6807 | 0.2047 | 0.0009 | 1.975 |
|  | Cardiac arrhythmia, irregular heartbeat and/or wear pacemaker | 1 | 0.2803 | 0.1384 | 0.0429 | 1.324 |
|  | Hypertension | 1 | 0.3353 | 0.1420 | 0.0183 | 1.398 |
|  | low DBP (<70 mmHg) | 1 | 0.2309 | 0.0980 | 0.0186 | 1.260 |
|  | Previous or current smoking | 1 | 0.2359 | 0.2474 | 0.3404 | 1.266 |
|  | Never drinking | 2 | 0.4978 | 0.3376 | 0.1406 | 1.645 |
|  | Obesity (BMI ≥30) | 0 |  |  |  |  |
|  | Overweight (BMI 25-20) | 2 | 0.5765 | 0.2923 | 0.0488 | 1.780 |
|  | Underweight or normal BMI (<25) | 5 | 1.1315 |  | 0.0002 | 3.100 |

## Supplementary Table 12. Association between vascular risk composite score and MCI risk from joint model

| **Effect** | **Estimate** | **SE** | **p-value** | **Odds ratio (OR)** |
| --- | --- | --- | --- | --- |
| **Intercept** | -5.4698 | 0.6725 | <0.001 | 0.004 |
| **Age 75-84** | 0.0552 | 0.1768 | 0.7551 | 1.057 |
| **Age ≥85** | -0.0555 | 0.3974 | 0.8889 | 0.946 |
| **Female** | -0.1796 | 0.2617 | 0.4927 | 0.836 |
| **= High school education** | -0.3244 | 0.4729 | 0.4928 | 0.723 |
| **> High school education** | -0.5882 | 0.5228 | 0.2607 | 0.555 |
| **Vascular Composite score*** | 0.2480 | 0.0335 | <0.001* | 1.281 |

*Estimates from model using composite score in replace of individual vascular predictors in the final joint model.

## Supplementary Table 13. Distribution of vascular composite scores by MCI status

| **Group** | **Vascular Composite score** | | | | | |
| --- | --- | --- | --- | --- | --- | --- |
|  | Mean | Min | Max | Q1 | Median | Q3 |
| **Non-MCI** | 10.4 | 0 | 34 | 6 | 9 | 14 |
| **MCI** | 14.5 | 1 | 31 | 9 | 14 | 19 |

## Supplementary Figure 3. Density plot of vascular composite score between MCI and non-MCI groups


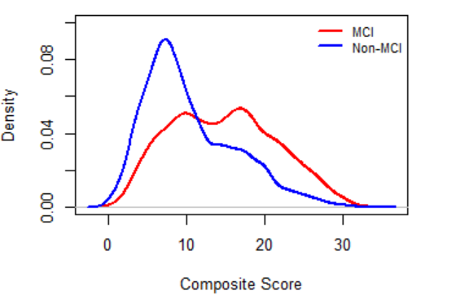


## Supplementary Figure 4. Vascular risk factor composite score contribution


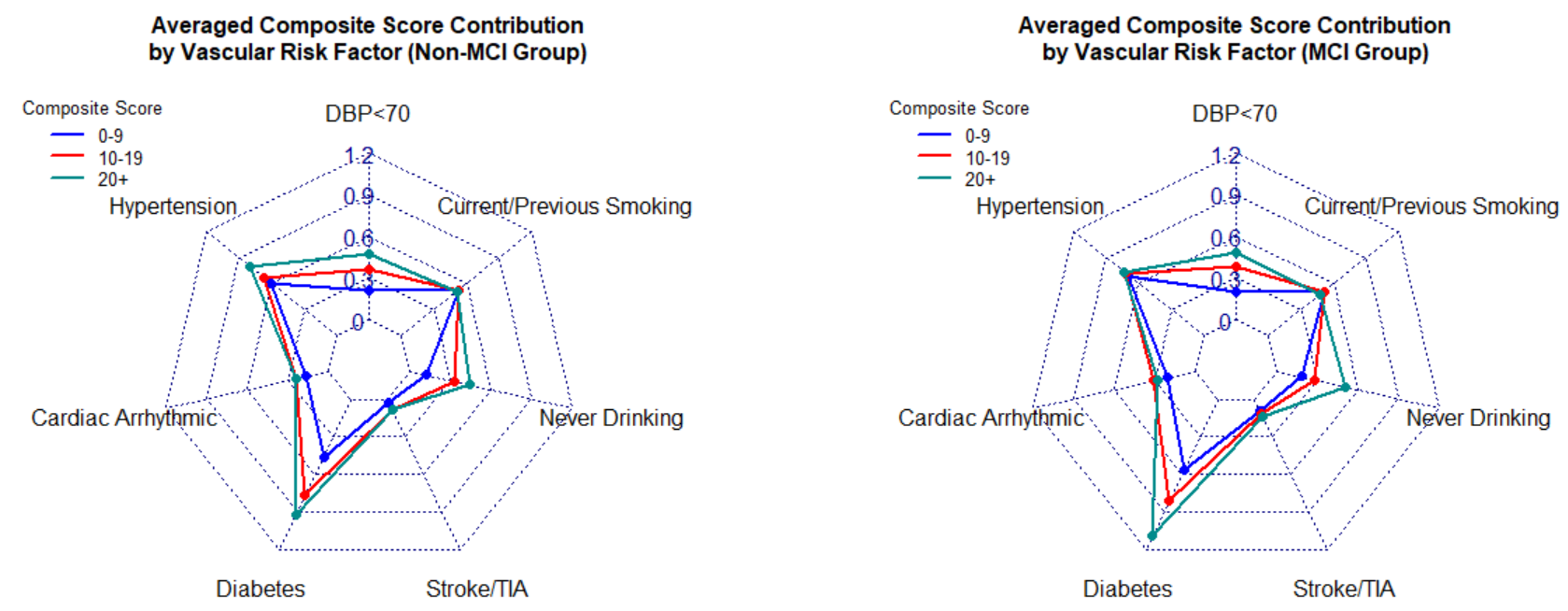


The radar chart on the left depicts the contribution of each vascular variable to the average composite score across composite score groups (0-9, 10-19, and ≥20) within the non-MCI group, based on joint modeling results excluding lab test variables. The radar chart on the right shows the corresponding contributions within the MCI group, also based on joint modeling results without lab test variables. Only variables included in the final reduced model are presented.
